# circCUL2 regulates gastric cancer malignant transformation and cisplatin resistance by modulating autophagy activation via miR-142-3p/ROCK2

**DOI:** 10.1186/s12943-020-01270-x

**Published:** 2020-11-05

**Authors:** Lei Peng, Huaiming Sang, Shuchun Wei, Yuanyuan Li, Duochen Jin, Xudong Zhu, Xuan Li, Yini Dang, Guoxin Zhang

**Affiliations:** 1 Department of Gastroenterology, First Affiliated Hospital of Nanjing Medical University, Nanjing, China; 2 Department of Gastroenterology, Renmin Hospital of Wuhan University, Wuhan, China; 3 Department of endocrinology, Children’s Hospital of Nanjing Medical University, Nanjing, China

**Keywords:** circCUL2, miR-142-3p, ROCK2, Gastric cancer, Cisplatin resistance, Autophagy

## Abstract

**Background:**

Circular RNAs (circRNAs) are a class of noncoding RNAs (ncRNAs) and can modulate gene expression by binding to miRNAs; further, circRNAs have been shown to participate in several pathological processes. However, the expression and biological function of circCUL2 in gastric cancer (GC) remains largely unknown.

**Methods:**

circRNA microarrays and quantitative real-time PCR (qRT-PCR) were used to identify differentially expressed circRNAs in GC tissues and cell lines. circCUL2 knockdown and overexpression were performed to indicate the functional role of circCUL2 in vitro and in vivo. The expression and regulation of circCUL2, miR-142-3p and ROCK2 were evaluated using fluorescence in situ hybridization (FISH), dual-luciferase assays, RNA pull-down assays, RNA immunoprecipitation (RIP) and rescue experiments. Furthermore, the regulation of cisplatin sensitivity and autophagy by circCUL2/miR-142-3p/ROCK2 was demonstrated by cellular apoptosis assays, western blot, immunofluorescence and transmission electron microscopy analyses.

**Results:**

The level of circCUL2, which is stable and cytoplasmically localized, was significantly reduced in GC tissues and cells. Overexpressed circCUL2 inhibited malignant transformation in vitro and tumorigenicity in vivo. In the AGS and SGC-7901 cell lines, circCUL2 sponged miR-142-3p to regulate ROCK2, thus modulating tumor progression. Furthermore, in the AGS/DDP and SGC-7901/DDP cell lines, circCUL2 regulated cisplatin sensitivity through miR-142-3p/ROCK2-mediated autophagy activation.

**Conclusion:**

circCUL2 may function as a tumor suppressor and regulator of cisplatin sensitivity through miR-142-3p/ROCK2-mediated autophagy activation, which could be a key mechanism and therapeutic target for GC.

**Supplementary information:**

**Supplementary information** accompanies this paper at 10.1186/s12943-020-01270-x.

## Introduction

Gastric cancer (GC) is the fifth most common cancer and ranks as the third major cause of cancer-related death globally [1, 2]. Due to the lack of specific symptoms and diagnostic markers in early stage GC, GC patients are always diagnosed at an advanced stage [3, 4]. Although treatments such as molecular-targeted therapy have emerged, surgical resection and adjuvant chemotherapy still serve as the main treatments for GC [5]. Chemotherapy is a first-line regimen with a poor curative effect due to chemoresistance, which develops by genetic and epigenetic modifications, signaling pathway alterations or cell metabolism disorders [6]. Therefore, further investigation of the mechanism of carcinogenesis and chemoresistance in GC is of great concern for early diagnosis and for improving the prognosis and survival of patients with advanced GC.

Circular RNA (circRNA) is a newly discovered type of noncoding RNA (ncRNA) characterized by a special single-stranded closed loop lacking both 5′-3′ polarity and a polyadenylated tail [7]. circRNA expression was found to be specific in diverse cell lines and tissue types, implying that circRNA has multiple functional roles in biological and pathological processes [8, 9]. An increasing number of studies have shown that circRNAs are differentially expressed in various cancers, such as breast cancer, oral squamous cell carcinoma and gastric cancer [10–12]. Studies have shown that circRNAs participate in various biological processes, such as proliferation, migration, apoptosis and the cell cycle, and thus participate in tumorigenesis and chemoresistance. Additionally, autophagy, which participates in carcinogenesis and chemoresistance, could be modulated by circRNA [13–15].

In this study, we found that circCUL2 was downregulated in GC tissues and cell lines. circCUL2 (circbase ID: hsa_circ_0000234), derived from back-splicing of the CUL2 mRNA (from exon 2 to exon 4), is located on chromosome 10:35,349,801-35,360,267 and is 339 nucleotides (nt) in length. It suppressed the proliferation, migration and invasion of GC cells. Furthermore, circCUL2 was downregulated in cisplatin-resistant GC cell lines and modulated cisplatin sensitivity. circCUL2 may be involved in tumorigenesis and chemoresistance by competitively binding to miR-142-3p and by modulating ROCK2 expression and autophagy activation.

## Methods

### Clinical specimens

Microarray data on 5 paired GC patients (GSE100170) [16] were downloaded from the GEO database and analyzed by R software. One hundred pairs of GC tissues and paired normal tissues were obtained from patients who underwent surgery at Jiangsu Province Hospital between 2011 and 2017. None of the patients received chemotherapy or radiotherapy before surgery. All the sample diagnoses were confirmed through pathological analysis; then, the samples were frozen in liquid nitrogen for 15 min and stored at − 80 °C until use. Blood samples were collected from 48 GC patients and 48 healthy controls (with no diagnosis of cancer). Informed consent was obtained from patients involved in the study. Tumors were staged according to the tumor-node-metastasis (TNM) staging system of the International Union Against Cancer (v.8, 2016). The protocols of this study were approved by the Ethics Committee of First Affiliated Hospital of Nanjing Medical University.

### Cell culture and transfection

Human GC cell lines (AGS, SGC-7901, MKN-45, and BGC-823) and the normal human gastric epithelial cell line GES-1 were purchased from the American Type Culture Collection (ATCC, USA). All these cells were cultured in RPMI 1640 medium (GIBCO, Brazil) supplemented with 10% fetal bovine serum (FBS; GIBCO, Brazil) and incubated at 37 °C in a humidified atmosphere with 5% CO_2_. AGS and SGC-7901 cells were transfected with Lipofectamine 2000 (Invitrogen, Carlsbad, CA, USA). After transfection for 24 h, cells were harvested for further investigation. The sequences used are listed in Additional file 1: Table S1. We synthesized human circCUL2 cDNA and subsequently cloned it into the pcDNA3.1 vector (Thermo Fisher, USA) to construct the circCUL2 overexpression plasmid.

### RNase R digestion and actinomycin D assay

A total of 3 μg of RNA was incubated with 20 U/μL RNase R (Epicentre Biotechnologies) for 15 min at 37 °C to confirm the circRNA characteristics. AGS and SGC-7901 cells were treated with actinomycin D for 0 h, 4 h, 8 h, 12 h and 24 h before RNA extraction. circCUL2 and CUL2 were detected in these two cell types.

### Sanger sequencing

The amplification products of circRNAs were inserted into a T-vector for Sanger sequencing by Tsingke (Nanjing, China). The primer (Invitrogen, Shanghai, China) was designed to confirm the back-splice junction of circCUL2.

### RNA extraction and quantitative real-time PCR (qRT-PCR)

Total RNA was isolated from clinical specimens or cell lines with TRIzol reagent (Invitrogen, Carlsbad, CA, USA). RNA (500 ng) was reverse transcribed into cDNA following the protocol of the PrimeScript RT Reagent Kit (TaKaRa Bio, Inc., China). Real-time quantitative PCR was used to detect the expression levels of circCUL2, miR-142-3p, and ROCK2. The circRNA and mRNA levels were normalized to that of GAPDH. The miRNA level was normalized to that of U6. The PCR primer sequences were synthesized by Tsingke (Nanjing, China) and are listed in Additional file 1: Table S2. RNA expression fold changes were determined with the 2^−ΔCt^ method.

### Isolation of nuclear and cytoplasmic fractions

Cytoplasmic and nuclear fractions were prepared using NE-PER Nuclear and Cytoplasmic Extraction Reagents (Thermo Scientific, USA) according to the manufacturer’s protocol. GC cells were lysed on ice for 10 min in Lysis Buffer J supplemented with protease inhibitors. After centrifugation at 14,000×g for 3 min, the resulting supernatant and sediment were collected as the cytoplasmic and nuclear fractions, respectively. RNA was extracted from each fraction by Buffer SK and washed with a wash solution. qRT-PCR was used to test the RNA expression of several RNAs.

### Fluorescence in situ hybridization (FISH)

Specific probes against circCUL2 and miR-142-3p were used for fluorescence in situ hybridization. The FISH procedures were conducted following the manufacturer’s instructions (GenePharma, Shanghai, China). Briefly, after fixation with 4% paraformaldehyde (PFA) for 15 min at room temperature, cells were washed twice with PBS, and were then mixed with 70%, 95 and 100% ethanol overnight at 4 °C. Hybridization of cells was carried out at 37 °C overnight in a dark moist chamber. After washing three times in saline-sodium citrate (SSC) buffer for 5 min and incubating in blocking buffer (1% BSA and 3% normal goat serum in PBS) for 1 h, cells were incubated with an HRP-conjugated anti-biotin antibody at 4 °C overnight. Finally, the cells were photographed using a fluorescence microscope (Olympus BX53, Olympus America, Inc., Center Valley, PA, USA).

### Protein extraction and western blot analysis

Radioimmunoprecipitation assay (RIPA) buffer was used for protein extraction. The supernatants from cell lysates were run on 10% acrylamide gels by SDS-PAGE and then transferred to a polyvinylidene difluoride membrane (Millipore). Antibodies against ROCK2 (1:1000, #ab71598, Abcam), LC3 (1:1000, #12741, Cell Signaling Technology), p62 (1:1000, #ab56416, Abcam), and Beclin1 (1:1000, #ab210498, Abcam) and an HRP-conjugated secondary antibody (1:2000) were employed for western blotting. A chemiluminescence western blotting detection system (Bio-Rad) was used for protein detection.

### Immunohistochemistry (IHC)

Tumor tissues were first fixed in 4% paraformaldehyde and embedded in paraffin, and 5-μm-thick sections were cut. Sections were blocked with 10% goat serum and incubated with an anti-ROCK2 (1:1000, #ab71598, Abcam) antibody at 4 °C overnight. Finally, images were acquired for analysis.

### Cell proliferation assay

Cell counting kit-8 (CCK-8, Dojindo, Osaka, Japan) was used to measure the proliferative capacity of GC cell lines. AGS and SGC-7901 cells were seeded in 96-well plates. Each well was treated with 10 μL of CCK-8 reagent, followed by incubation at 37 °C for another 1 h before the detection of absorbance at 450 nm by use of a microplate reader (Bio-Rad, CA, USA). Cell proliferation was observed at different times (6, 24, 48, 72 and 96 h). All experiments were performed in triplicate.

### 5-Ethynyl-2′-deoxyuridine (EdU) incorporation assay

A Cell-Light EdU DNA Cell Proliferation Kit (RiboBio, Guangzhou, China) was used to perform the EdU assay. After incubation with 50 mM EdU for 2 h, the AGS and SGC-7901 cells were fixed in 4% paraformaldehyde and stained with Apollo Dye Solution, and then Hoechst 33342 was used to identify the nuclei. Then, the proliferation-positive cells were photographed and counted under an Olympus FSX100 microscope (Olympus, Tokyo, Japan).

### Colony formation assay

GC cell lines (AGS and SGC-7901) resuspended to 1× 10^3^ cells/mL were seeded in 6-well plates. After incubation at 37 °C for 14 days, cells were stained with 0.1% crystal violet and 20% methanol, and then the cell colonies were counted.

### Migration and invasion assay

Transwell assays were used for migration and invasion assays. For invasion assays, the lower chambers were precoated with 100 μL of Matrigel (BD Bioscience, San Jose, CA, USA) for 30 min before the addition of medium to the chambers. The transfected GC cells (1 × 10^6^ cells/mL) were resuspended in RPMI 1640 medium. The upper chamber contained 100 μL of cell suspension medium, and 600 μL of complete medium was added to the bottom chamber. After incubating at 37 °C with 5% CO_2_ for 24 h, cells were fixed with 4% paraformaldehyde and stained with 0.1% crystal violet solution. The cells that passed through the filter were photographed and counted by inverted fluorescence microscopy (Leica Microsystems GmbH, Wetzlar, Germany) in five randomly selected fields.

### Wound healing assay

Cells were cultured in 6-well plates for 24 h, and a sterile pipette tip (200 μL) was used to scratch the monolayer. The cells were washed with PBS three times and were incubated in serum-free DMEM. Cell migration was photographed 24 h after scratching by an inverted microscope (Olympus, Japan), and the total wound area was analyzed using ImageJ software to assess the cell migration capacity.

### Mouse xenograft model

Six-week-old female nude mice were purchased from the Laboratory Animal Center of Nanjing Medical University and maintained under pathogen-free conditions. Nude mice were subcutaneously injected in the inguinal region with 5× 10^6^ GC cells (5 mice per group). Tumor volumes were measured every 4 days and calculated using the following formula: volume = length × (width/2)^2^. After 28 days, all the mice were sacrificed, and then tumors were resected and collected. To test chemosensitivity, one week after injection, cisplatin (5 mg/kg) in PBS was intraperitoneally injected into the mice three times per week. The xenograft tumors were harvested after 4 weeks. All experiments were approved by the Ethics Committee of the First Affiliated Hospital of Nanjing Medical University.

### Luciferase reporter assay

GC cells were seeded in 96-well plates and then cotransfected with circCUL2/ROCK2 wild-type or mutant plasmids and miR-142-3p mimics or miR-NC using Lipofectamine 2000. After 48 h of incubation, the luciferase activity was measured according to the manufacturer’s instructions (Promega, Madison, WI, USA). Each experiment was repeated at least three times.

### RNA immunoprecipitation (RIP)

The RIP assay was carried out by using a Magna RIP RNA Binding Protein Immunoprecipitation Kit (Millipore) according to the manufacturer’s instructions. The antibodies against AGO2 and IgG used for the RIP assays were purchased from Abcam (ab5072, rabbit polyclonal antibody, Cambridge, MA, USA).

### RNA pull-down assay

The biotinylated circCUL2 probes were designed and synthesized by GenePharma (Shanghai, China). The circCUL2 probe was first incubated with C-1 magnetic beads (Life Technologies, Waltham, MA, USA) for 2 h at 25 °C to coat the beads with the probe. After the cells were harvested and lysed, the lysates were incubated with the circCUL2 probe or oligo probe at 4 °C overnight. The RNA-beads complexes were extracted with a RNeasy Mini Kit. The abundances of circCUL2 and miR-142-3p were evaluated by qRT-PCR.

### Apoptosis analysis

Based on the manufacturer’s guidelines, cell apoptosis was measured by a flow cytometer (FACSCalibur, BD, USA). After 24 h of treatment, the cells were washed, resuspended, and then stained with FITC and PI, after which flow cytometry was used to analyze the apoptosis rate of cells treated under different conditions. The cell apoptosis data were analyzed by FlowJo V10 software (Tree Star, San Francisco, CA, USA). Each experiment was performed more than three times.

### Cell viability analysis

Cells with or without transfection were seeded in 96-well plates at 5 × 10^3^ cells/well for 24 h and then treated with cisplatin (DDP; 0, 1, 2.5, 5, 7.5, 15, 25 μg/mL), 5-fluorouracil (5-FU; 0, 1.5, 3, 6, 12, 25, and 45 μg/mL), doxorubicin hydrochloride (DOX; 0, 0.1, 0.5, 1, 2.5, 5, and 10 μg/mL), and mitomycin C (MMC; 0, 0.1, 0.5, 1, 2.5, 5, and 10 μg/mL) for 48 h. The cell viability was detected using an MTT Assay Kit (Sigma, St. Louis, MO, USA) to measure the cytotoxicity of cisplatin. The half maximal inhibitory concentration (IC50) was determined based on the absorbance at 490 nm as measured in a microplate reader (Bio-Rad, CA, USA).

### Immunofluorescence

Cells in confocal dishes were treated for 48 h, fixed with 4% paraformaldehyde, treated with Triton X-100, blocked with goat serum, incubated with a primary antibody (against LC3 (1:100, #12741, Cell Signaling Technology)) and a secondary antibody, stained with DAPI, and viewed using a confocal laser scanning microscope (ZEISS, Germany).

### Transmission electron microscopy (TEM)

Cells were fixed in 2% glutaraldehyde overnight at 4 °C, incubated in 1% osmium tetroxide for 1 h at 4 °C, dehydrated in graded ethanol, saturated in graded acetone, cut into 50-nm ultrathin sections, stained with lead citrate and viewed using JEM-1010 TEM (JEOL, Japan).

### Statistical analysis

Data are reported as the means ± standard deviations (SDs), and GraphPad Prism (version 7.0) was used for the general statistical analysis. Student’s t-test was used to analyze the differences between two groups. The chi-square test was used to investigate the significance of the correlation of circCUL2 expression with the clinicopathological features. The survival curves were calculated by the Kaplan-Meier method and compared with the log-rank test. A *P*-value less than 0.05 was considered statistically significant.

## Results

### The expression and characteristics of circCUL2 in GC tissues and cells

**Fig. 1:**
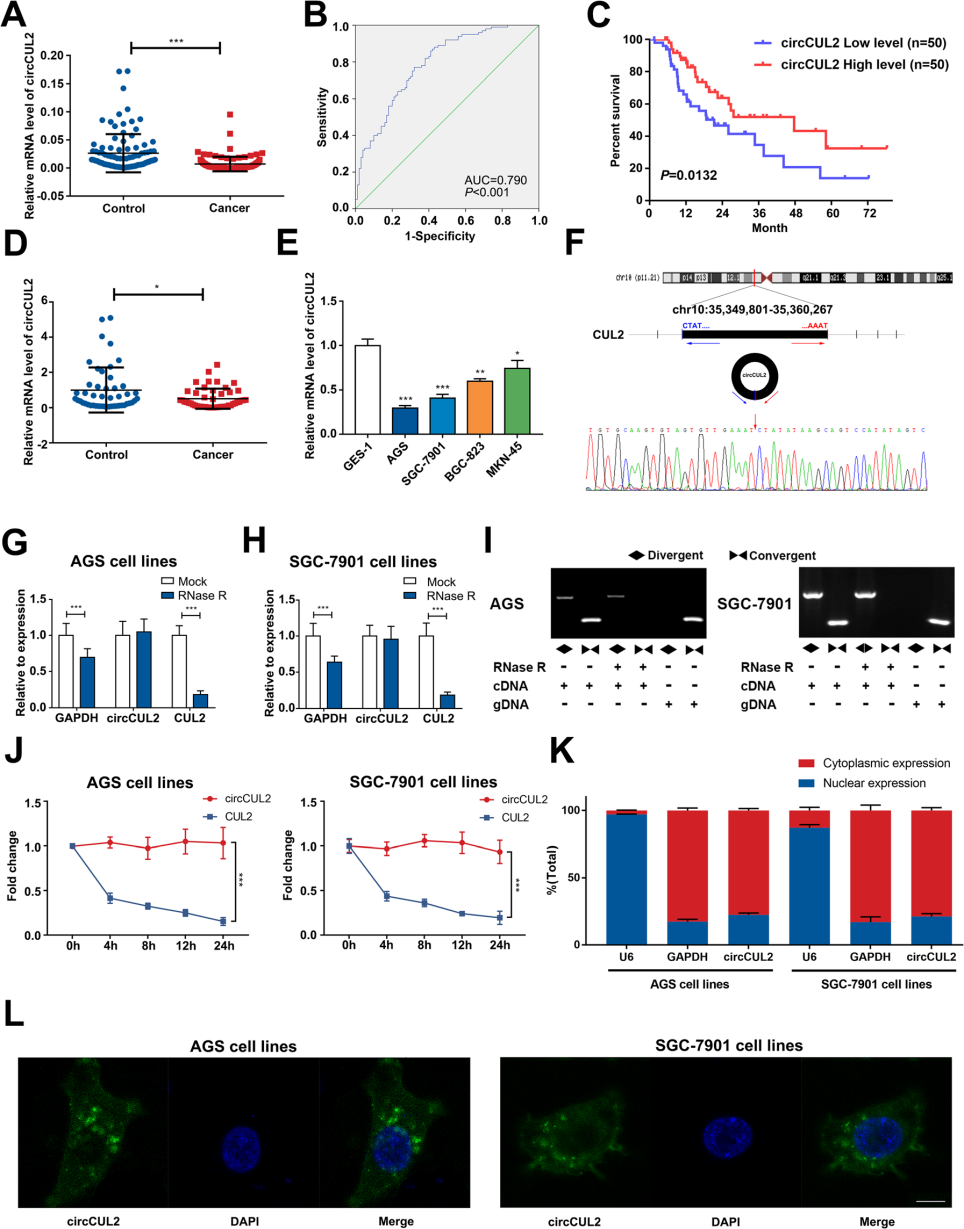
The expression and characteristics of circCUL2 in GC tissues and cells. **a**: qRT-PCR of circCUL2 expression in GC tissues. **b**: The ROC curve to evaluate the diagnostic value of circCUL2. **c**: Kaplan-Meier analysis of the correlation between circCUL2 expression and overall survival in 100 GC patients. **d**: Relative circCUL2 levels in serum from GC patients and healthy controls without GC (*n* = 48). **e**: The expression of circCUL2 in GC cells. **f**: Sanger sequencing of circCUL2. The arrows represent splicing sites. G and H: qRT-PCR determining the abundance of circCUL2 and linear CUL2 in the AGS (**g**) and SGC-7901 (**h**) cell lines after RNase R treatment. **i**: qRT-PCR products of linear and circular products amplified with convergent and divergent primers with and without RNase R treatment. **j**: qRT-PCR analysis of circCUL2 and CUL2 after actinomycin D treatment. **k**: qRT-PCR of separated nuclear and cytoplasmic fractions. **l**: FISH for circCUL2 localization in AGS and SGC-7901 cell lines

The microarray GSE100170 of 5 paired GC patients was applied to explore several circRNAs differentially expressed between GC tissues and adjacent tissues (Additional file 2: Fig. S1A and B). GO and KEGG analyses based on the host genes of differentially expressed circRNAs were conducted and showed that these genes were enriched in tumorigenesis, drug resistance, and digestive diseases (Additional file 2: Fig. S1C and D). Based on the differentially expressed genes and the results of the GO and KEGG analyses, circCUL2 was selected for further research. The expression of circCUL2 was significantly lower in GC tissues than in paired noncancerous tissues (Fig. 1a). The clinicopathological features were analyzed based on the expression of circCUL2 in GC tissues (Table 1). In addition, low expression of circCUL2 was correlated with late-stage GC (stage III + IV), lymph node metastasis and poor GC differentiation (Additional file 2: Fig. S2A-C). There was no significant difference in circCUL2 expression based on other clinical parameters, including gender, age, tumor depth and tumor size (Additional file 2: Fig. S2D-G). Moreover, circCUL2 expression was not correlated with the *Helicobacter pylori* infection status or Lauren classification based on data from part of the patients (Additional file 2: Fig. S2H-I). Furthermore, receiver operating characteristic (ROC) analysis, which investigated the diagnostic value of circCUL2 in GC, showed that the area under the ROC curve (AUC) was 0.790 (*P* < 0.001) (Fig. 1b). Kaplan-Meier analysis revealed that GC patients with low circCUL2 levels had poor overall survival (*P* = 0.0132) (Fig. 1c). Next, via qRT-PCR, significantly decreased circCUL2 levels were found in GC serum compared with normal serum (Fig. 1d). In addition, the circCUL2 level was decreased in the AGS and SGC-7901 cell lines, which were selected for further research (Fig. 1e).

**Table 1:** Association between circCUL2 expression and clinicopathological characteristics in gastric cancer

Characteristics	***n***	High expression	Low expression	***P***
**Gender**				
**Female**	32	14	18	0.8862
**Male**	68	36	32	
**Age(y)**				
**≤ 60**	41	17	24	0.8529
**> 60**	59	33	26	
**Tumor size (cm)**				
**≥ 3**	65	34	31	0.8437
**< 3**	35	16	19	
**Histological type**				
**Well differentiated**	39	28	11	< 0.001***
**Poorly differentiated**	61	22	39	
**Clinical stage**				
**I-II**	37	22	15	< 0.001***
**III-IV**	63	28	35	
**Tumor depth**				
**T1-T2**	23	10	13	0.6132
**T3-T4**	77	40	37	
**Lymph node metastasis**				
**No**	19	13	6	< 0.001***
**Yes**	81	37	44	
**Lauren classification**				
**Intestinal**	49	27	22	0.6275
**Diffuse**	21	12	9	
**Uncertain**	30	11	19	
**Helicobacter Pylori**				
**Positive**	22	5	17	0.0524
**Negative**	41	22	19	
**Uncertain**	37	23	14	

Subsequently, we verified the sequence of the circCUL2 qRT-PCR product amplified by divergent primers with Sanger sequencing, and the target region was the head-to-tail splicing site, and the sequence was consistent with that in the circBase database (Fig. 1f). Then, cDNA and genomic DNA (gDNA) with or without RNase R treatment were amplified by convergent primers or divergent primers to amplify linear or circular CUL2. The results showed that circCUL2, which was observed in only cDNA amplified by divergent primers but not in gDNA, could resist RNase R treatment (Fig. 1g-i). The linear CUL2 amplified by convergent primers was digested by RNase R (Fig. 1g-i). Moreover, the linear transcript of CUL2 demonstrated a shorter half-life than circCUL2 in AGS and SGC-7901 cell lines treated with the transcription inhibitor actinomycin D (Fig. 1j). qRT-PCR analysis of nuclear and cytoplasmic RNA and FISH against circCUL2 demonstrated that circCUL2 preferentially localized in the cytoplasm (Fig. 1k-l). Collectively, these results revealed that circCUL2, which is stable and localized mainly in the cytoplasm, may participate in the occurrence and development of GC.

### circCUL2 suppresses the malignant transformation of GC cells

**Fig. 2:**
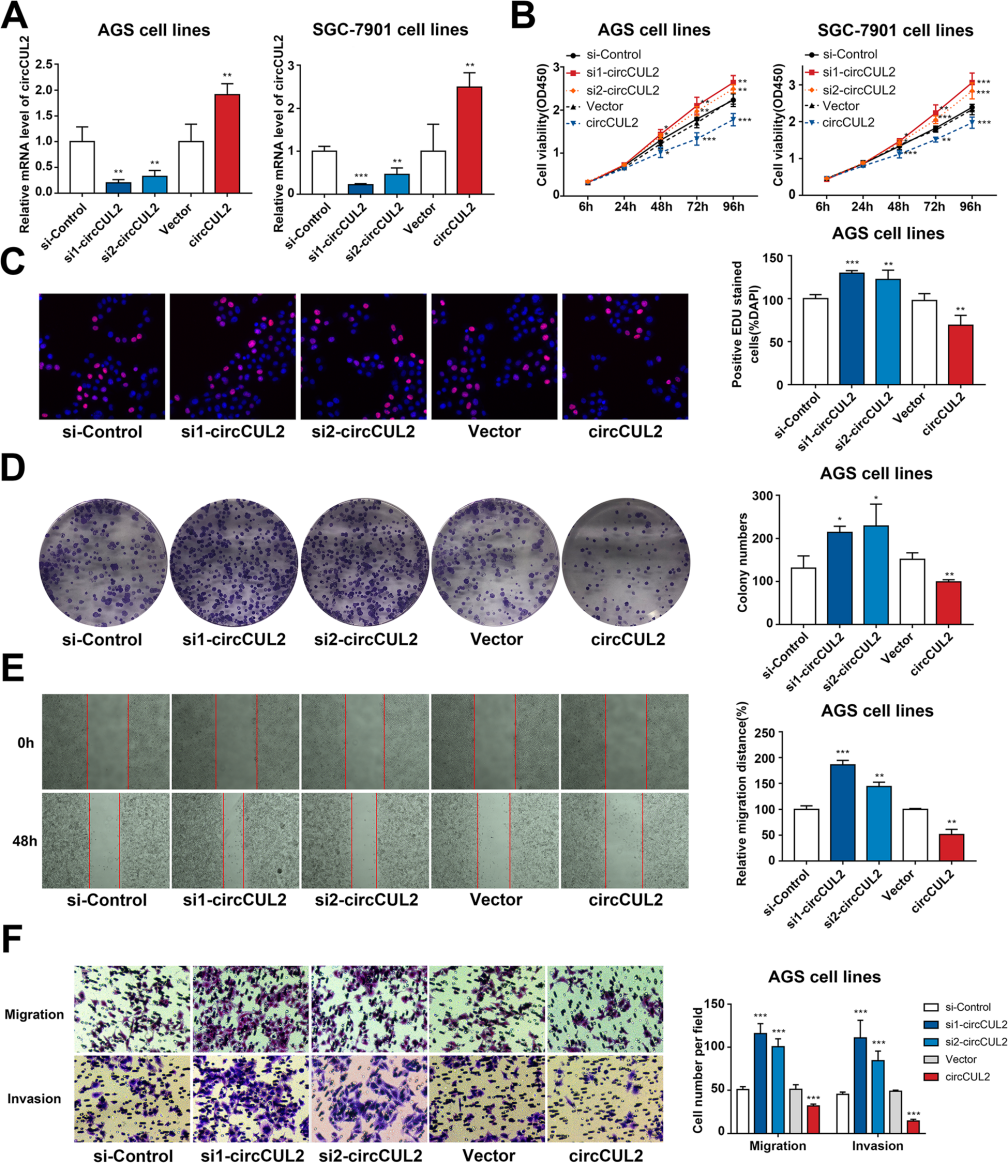
circCUL2 suppresses the malignant transformation of GC cells. **a**: qRT-PCR analysis of circCUL2 mRNA expression after treatment with two siRNAs and an overexpression plasmid. **b**: Assessment of the proliferation of AGS and SGC-7901 cells transfected with circCUL2-specific siRNA or an overexpression plasmid by a CCK-8 assay. **c-d**: Assessment of AGS cell proliferation by EdU **c** and colony formation assays **d**. **e**: Wound healing assay to detect the effect of circCUL2 on cell migration. **f**: Decreased or increased circCUL2 overexpression regulated the migration and invasion of AGS cells

To further explore the functional effects of circCUL2 on GC cells, two siRNAs were designed to target the junction site (Additional file 2: Fig. S3A). RNA interference and overexpression plasmids were utilized for the following experiments (Fig. 2a). CCK-8 and EdU assays were used to verify that the proliferative capacity of GC cells was enhanced by circCUL2 downregulation (Fig. 2b-c, Additional file 2: Fig. S3B). The effects on colony formation were also enhanced by circCUL2 inhibition (Fig. 2d, Additional file 2: Fig. S3C). In addition, cell migration was examined by Transwell and wound healing assays and was found to be increased by circCUL2-specific siRNA (Fig. 2e and f, Additional file 2: Fig. S3D-E). Invasion, which was suppressed in circCUL2-overexpressing cells, was promoted in cells with decreased circCUL2 expression (Fig. 2f, Additional file 2: Fig. S3E). Overexpression of circCUL2 had the opposite effect: it appeared to inhibit cell proliferation, migration and invasion (Fig. 2b-f).

### circCUL2 functions as a miR-142-3p sponge

**Fig. 3:**
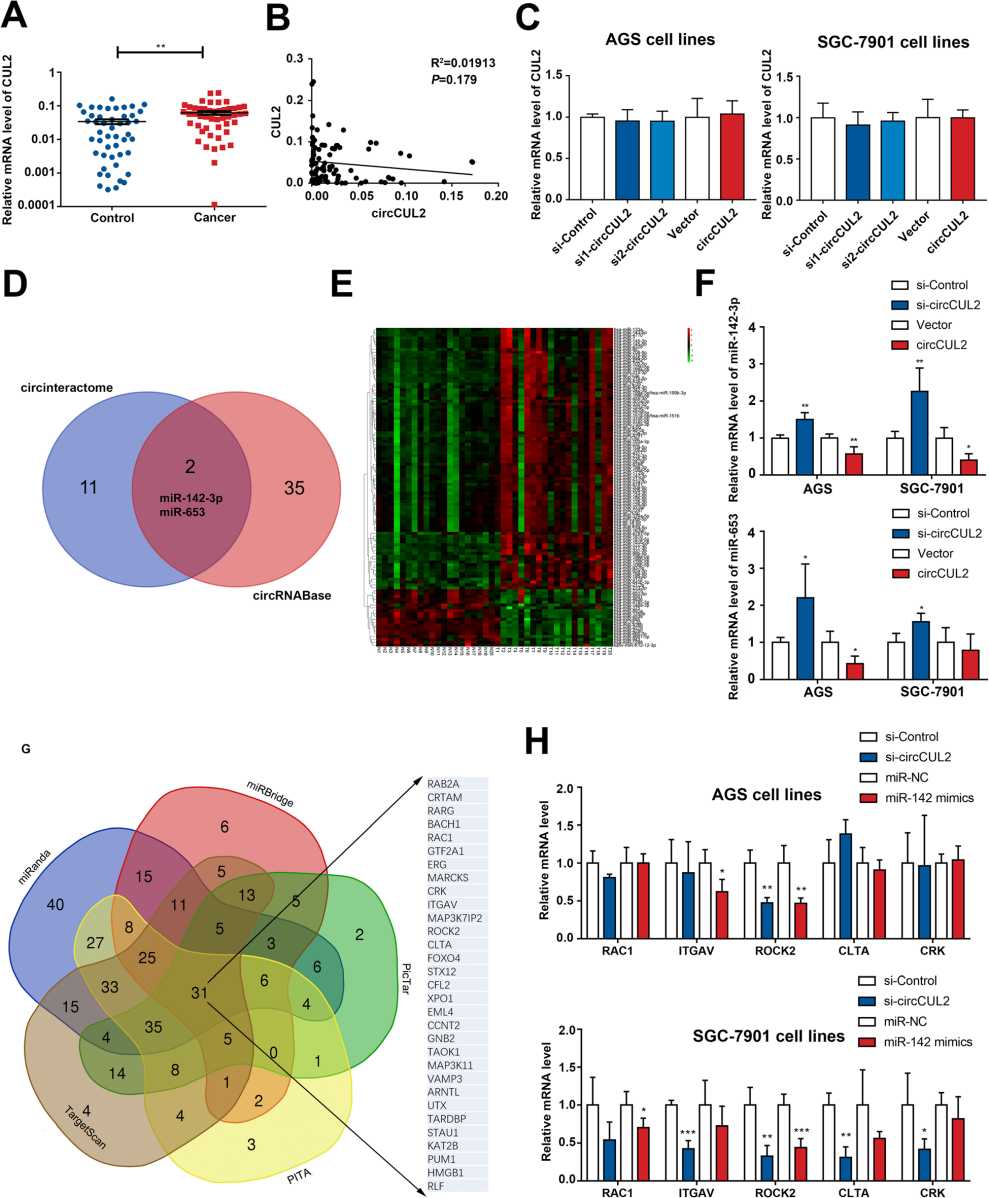
circCUL2 functions as a miR-142-3p sponge. **a**: The expression of linear CUL2 in GC tissues. **b**: The correlation of circCUL2 and CUL2 (*P* = 0.179). **c**: Neither knockdown nor overexpression of circCUL2 modulated linear CUL2 expression in GC cells. **d**: CircInteractome and circRNABase were used to predict the potential target miRNAs that bind to circCUL2. **e**: Heatmap of miRNA sequencing data (GSE89143). **f**: The expression of miR-142-3p and miR-653 in AGS and SGC-7901 cells transfected with circCUL2-specific siRNA or overexpression plasmids. **g**: The target genes of miR-142-3p predicted by miRanda, miRBridge, PicTar, PITA and TargetScan. **h**: The expression of screened miR-142-3p target genes (RAC1, ITGAV, ROCK2, CLTA and CRK) in AGS and SGC-7901 cells transfected with circCUL2-specific siRNA or miR-142-3p mimics

However, the mechanism by which circCUL2 participates in GC progression remains unknown. The cytoplasmic location of circCUL2 indicated that circCUL2 may be involved in the development of GC at the posttranscriptional level. To examine the alternative splicing mechanism, linear CUL2 expression was detected in GC tissues. The qRT-PCR results showed that CUL2 was increased in GC tissues but had no correlation with circCUL2 (Fig. 3a-b). In addition, neither suppression nor overexpression of circCUL2 changed the mRNA expression level of linear CUL2 (Fig. 3c). Moreover, the results of the CCK-8 and EdU assays revealed that decreased expression of CUL2 suppressed cell proliferation (Additional file 2: Fig. S4A-C). However, the results of the Transwell migration and invasion assays demonstrated that inhibition of CUL2 expression did not affect cell migration and invasion (Additional file 2: Fig. S4D). Moreover, overexpression of CUL2 had the opposite effect (Additional file 2: Fig. S4B-D).

After excluding the alternative splicing mechanism, we next investigated the ability of circCUL2 to bind to miRNAs. The tools CircInteractome and circRNABase were used to predict the potential target miRNAs that could bind to the circCUL2 sequence (Fig. 3d). Combined with the miRNA microarray of 20 paired GC tissues (Fig. 3e), miR-142-3p and miR-653 were selected. Only the level of miR-142-3p was increased or decreased in cells treated with circCUL2 siRNA or circCUL2 overexpression plasmids, respectively (Fig. 3f). Then, the target genes of miR-142-3p, which were predicted by miRanda, miRBridge, PicTar, PITA and TargetScan, were screened by functional analysis based on the NCBI database (Fig. 3g). Only ROCK2 was found to be regulated by circCUL2 and miR-142-3p (Fig. 3h). Based on these results, we speculated that circCUL2 may function as a miR-142-3p sponge to regulate ROCK2.

### circCUL2, miR-142-3p and ROCK2 expression in vivo

**Fig. 4:**
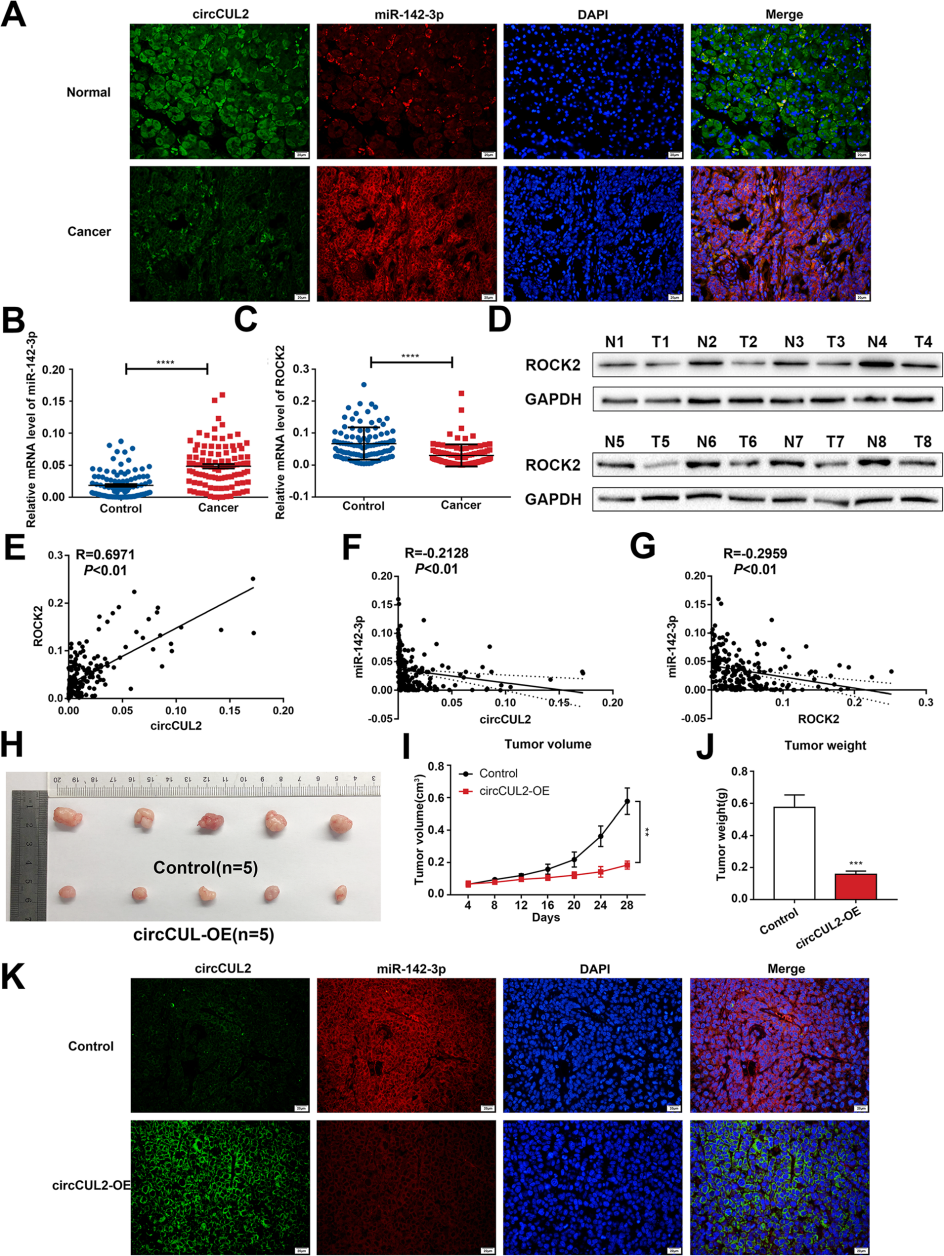
circCUL2, miR-142-3p and ROCK2 expression in vivo. **a**: FISH for circCUL2 and miR-142-3p analysis in GC and normal tissues. **b** and **c**: The miRNA levels of miR-142-3p **b** and mRNA level of ROCK2 **c** in GC tissues detected by qRT-PCR. **d**: The protein level of ROCK2 in GC tissues detected by western blot. **e**: The positive correlation of circCUL2 expression with ROCK2 expression (*P* < 0.01). **f-g**: The negative correlation of miR-142-3p expression with circCUL2 expression (*P* < 0.01) **f** and ROCK2 expression (*P* < 0.01) **g**. **h**: Nude mice were subcutaneously injected with SGC-7901 cells overexpressing circCUL2. **i**: Tumor volumes were measured in mice that received subcutaneous injections. **j**: After the mice were sacrificed, tumors were weighed separately. **k**: FISH analysis of circCUL2 and miR-142-3p in mouse tumor tissues

To investigate the roles of circCUL2, miR-142-3p and ROCK2 in vivo, FISH was conducted for circCUL2 and miR-142-3p in GC tissues. The results revealed that circCUL2 and miR-142-3p were colocalized and had opposite expression (Fig. 4a). The miRNA level of miR-142-3p was significantly upregulated (Fig. 4b), while the mRNA and protein levels of ROCK2 were significantly decreased in GC tissues (Fig. 4c-d, Additional file 2: Fig. S5A). In addition, higher expression of miR-142-3p was correlated with late-stage GC (stage III + IV), lymph node metastasis and poor GC differentiation (Additional file 2: Fig. S5B-H). Moreover, a significant inverse correlation was found between miR-142-3p and the circCUL2 or ROCK2 expression levels, while a positive correlation was found between circCUL2 and ROCK2 expression (Fig. 4e-g).

Furthermore, cells stably transfected with circCUL2-OE or an empty vector were inoculated subcutaneously into nude mice, and these mice were monitored closely for tumor growth for 4 weeks. Our results illustrated that tumors derived from circCUL2-OE cells were significantly smaller than those derived from empty vector-transfected cells (Fig. 4h) in terms of both volume and weight (Fig. 4i-j). Then, FISH was used to detect circCUL2 and miR-142-3p, qRT-PCR was used to detect the circCUL2, miR-142-3p and ROCK2 mRNA levels, and IHC was used to detect the ROCK2 protein levels in tumors derived from nude mice. Colocalization and inverse expression levels of circCUL2 and miR-142-3p were observed based on FISH (Fig. 4k) and qRT-PCR (Additional file 2: Fig. S5I), while the increased expression of ROCK2 associated with circCUL2 overexpression was confirmed by qRT-PCR and IHC (Additional file 2: Fig. S5I-J).

### circCUL2 regulates malignant transformation through miR-142-3p/ROCK2 in vitro

**Fig. 5:**
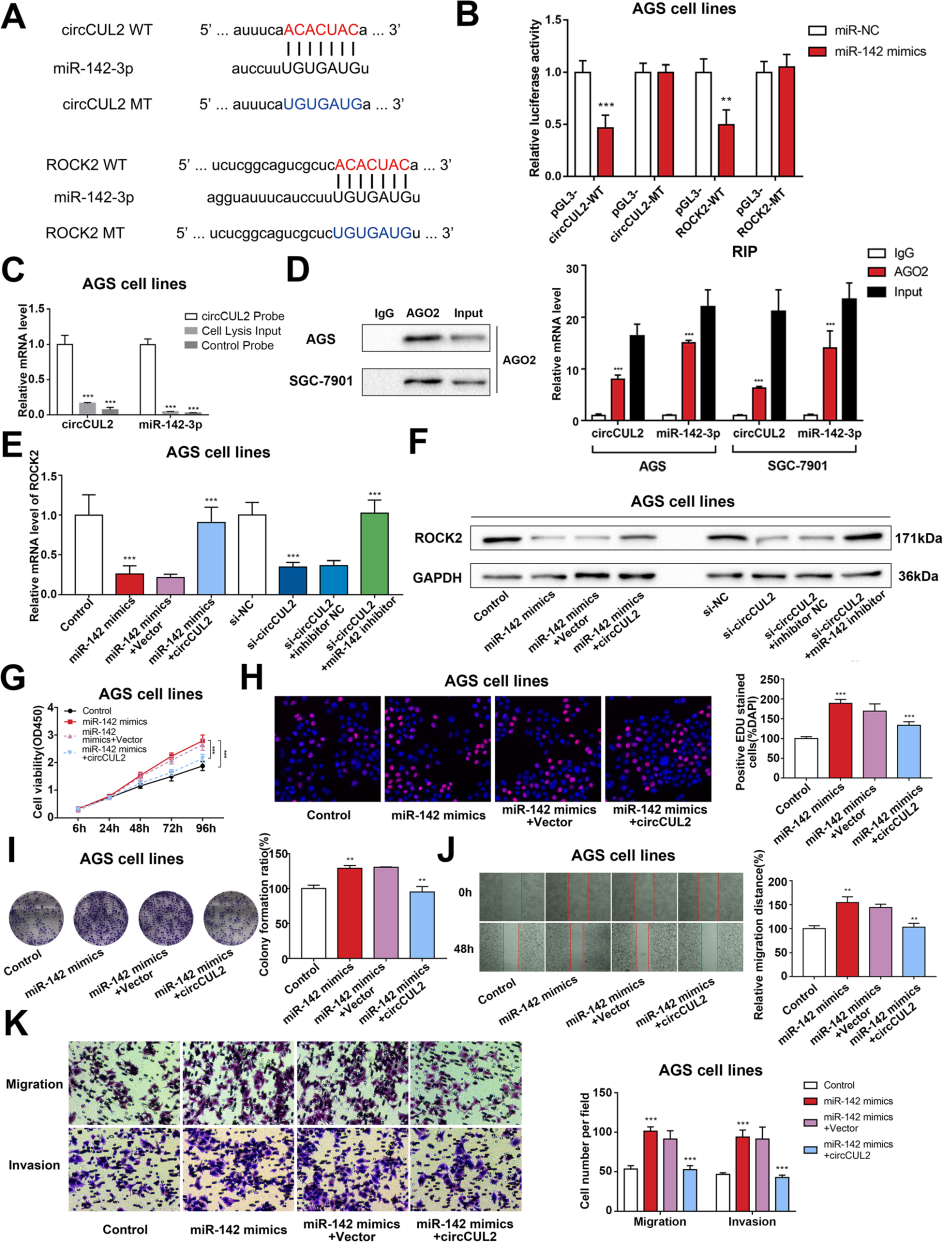
circCUL2 regulates malignant transformation through miR-142-3p/ROCK2 in vitro. **a**: The mutant and putative binding site of miR-142-3p with circCUL2 (upper) and ROCK2 (lower). **b**: A luciferase reporter assay was used to detect the binding of miR-142-3p to circCUL2 and ROCK2 in AGS cell lines. **c**: qRT-PCR of circCUL2 and miR-142-3p expression in AGS cell lysates pulled down and enriched with a circCUL2-specific probe. **d**: RIP with an anti-AGO2 antibody in AGS and SGC-7901 cell lines was used to detect the circCUL2 and miR-142-3p mRNA levels (right). The AGO2 protein level was determined by western blotting (left). **e** and **f**: Cotransfection of miR-142-3p mimics and circCUL2 overexpression plasmid or miR-142-3p inhibitor and circCUL2-specific siRNA to detect the mRNA **e** and protein **f** levels of ROCK2 in AGS cell lines. **g-k**: Cotransfection of miR-142-3p mimics and circCUL2 overexpression plasmid to investigate malignant transformation of cells with CCK-8 **g**, EdU **h**, colony formation **i**, wound healing **j** and Transwell **k** assays in the AGS cell line

Further research was conducted in vitro to investigate whether circCUL2/miR-142-3p/ROCK2 could regulate tumorigenesis and malignant transformation. The effect of individual circCUL2 overexpression plasmids and siRNAs, miR-142-3p mimics and inhibitors on the expression of miR-142-3p and ROCK2 was detected by qRT-PCR. miR-142-3p had an opposite expression pattern from circCUL2 and ROCK2, while ROCK2 expression exhibited the same trend as circCUL2 expression (Additional file 2: Fig. S6A-B). As expected, bioinformatics database analysis predicted that circCUL2 and ROCK2 could bind to miR-142-3p (Fig. 5a). The luciferase reporter assay results further confirmed that miR-142-3p could bind directly to a site in circCUL2 and the 3′-UTR of ROCK2 (Fig. 5b, Additional file 2: Fig. S6C). Next, circRNA pull-down experiments were conducted with a specific biotin-labeled circCUL2 probe. Then, we purified circCUL2-associated RNA and performed qRT-PCR to measure circCUL2 and miR-142-3p expression. The RNA pull-down results showed that, compared to the control group, circCUL2 and miR-142-3p were significantly enriched in circCUL2-specific probe pulled-down samples (Fig. 5c, Additional file 2: Fig. S6D). Next, we performed RIP with an anti-AGO2 antibody in the AGS and SGC-7901 cell lines. Our results showed that circCUL2 and miR-142-3p were significantly enriched, as they were precipitated by the anti-AGO2 antibody (Fig. 5d). Collectively, these results suggest that circCUL2 may act as a binding platform for miR-142-3p.

Moreover, the potential mechanisms by which circCUL2/miR-142-3p might regulate GC progression were explored. The decreases in the mRNA and protein levels of ROCK2, which were induced by miR-142-3p mimics or circCUL2 siRNA, were reversed by circCUL2 overexpression or transfection with a miR-142-3p inhibitor, respectively (Fig. 5e and f, Additional file 2: Fig. S6E-F). Additionally, miR-142-3p overexpression in AGS and SGC-7901 cells increased the proliferation rate (Fig. 5g-h, Additional file 2: Fig. S6G-H), migration ability, and invasion ability (Fig. 5j-k, Additional file 2: Fig. S6J-K). To further address whether circCUL2 performs its function by interacting with miR-142-3p, we cotransfected miR-142-3p mimics and circCUL2 expression plasmids into GC cells. The effects on GC cell growth and motility promotion induced by miR-142-3p were reversed in cells overexpressing circCUL2 (Fig. 5g-k, Additional file 2: Fig. S6G-K). These data reveal that circCUL2 inhibits GC cell growth and metastasis by sponging miR-142-3p.

### circCUL2 regulates the sensitivity of GC cells to cisplatin

To further verify the circCUL2/miR-142-3p/ROCK2 axis, we analyzed the miR-142-3p and ROCK2 levels in the TCGA database (Additional file 2: Fig. S7A-B). A negative correlation was found between miR-142 and ROCK2 (Additional file 2: Fig. S7C). However, there was no significant difference in the expression of either miR-142-3p or ROCK2 based on TNM stage (Additional file 2: Fig. S7D-E). Furthermore, the results of the CCK-8, EdU, Transwell migration and invasion assays revealed that decreased expression of ROCK2 rescued the cell proliferation, migration and invasion inhibited by miR-142-3p inhibitor (Additional file 2: Fig. S7F-I).

**Fig. 6:**
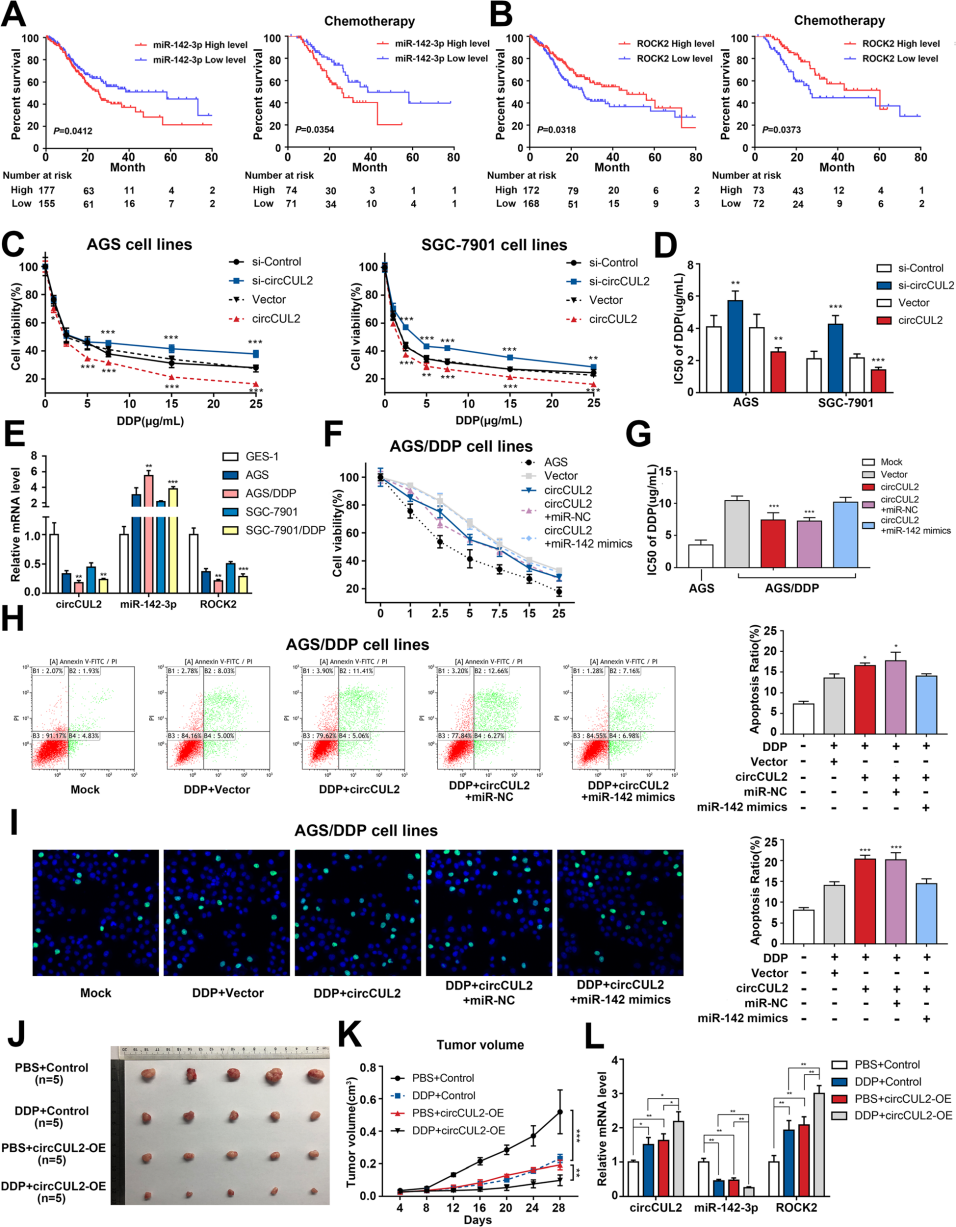
circCUL2 regulates the sensitivity of GC cells to cisplatin. **a**: Kaplan-Meier analysis of the correlation between miR-142-3p expression and DFS (left) or DFS with chemotherapy (right). **b**: The correlation between ROCK2 expression and DFS (left) or DFS with chemotherapy (right). **c**: Relative viability of circCUL2 overexpression plasmid- or siRNA-transfected cells exposed to cisplatin at the indicated concentrations for 48 h. **d**: The IC50 of AGS and SGC-7901 cell lines treated with cisplatin. **e**: The mRNA levels of circCUL2, miR-142-3p and ROCK2 in the GSE-1, AGS, AGS/DDP, SGC-7901 and SGC-7901/DDP cell lines. **f-i**: Cell growth **f**, IC50 **g**, apoptosis ((flow cytometry) **h**, and TUNEL assays **i** of AGS/DDP cell lines cotransfected with miR-142-3p mimics and circCUL2 overexpression plasmids. **j**: Xenograft tumors of sacrificed mice treated with or without cisplatin at the experimental endpoint. **k**: Tumor volumes of nude mouse tumors. **l**: The mRNA levels of circCUL2, miR-142-3p and ROCK2 in mice tumor tissues

Since miR-142-3p and ROCK2, downstream of circCUL2, could be relevant to GC prognosis [17, 18], we analyzed the TCGA database and found that higher expression of miR-142-3p and lower expression of ROCK2 resulted in shorter disease-free survival (DFS) or DFS combined with chemotherapy (Fig. 6a-b). These results indicated that circCUL2 might participate in the chemoresistance of GC cells. Therefore, cisplatin, 5-FU, DOX and MMC were screened to identify the drug to which sensitivity was most regulated by circCUL2. The results revealed that increased circCUL2 mostly inhibited the cell viability of cells treated with cisplatin, while the decreased circCUL2 mostly induced cisplatin resistance (Fig. 6c, Additional file 2: Fig. S8A-C). The half maximal inhibitory concentration (IC50) was also increased by circCUL2-specific siRNA and decreased by transfection of the circCUL2 overexpression plasmid (Fig. 6d).

To verify the hypothesis that circCUL2 regulates cisplatin sensitivity, we constructed the cisplatin-resistant AGS/DDP and SGC-7901/DDP cell lines. Compared to the corresponding normal GC cell line, cisplatin-resistant GC cell lines had significantly lower circCUL2 and ROCK2 expression and significantly higher miR-142-3p expression (Fig. 6e). Subsequently, the AGS/DDP and SGC-7901/DDP cell lines were treated with cisplatin after cotransfection of miR-142-3p mimics and circCUL2 expression constructs. The effects of proliferation suppression and apoptosis promotion induced by circCUL2 were reversed when the cells overexpressed miR-142-3p (Fig. 6f-i, Additional file 2: Fig. S9A-D).

In vivo, SGC-7901 cells stably overexpressing circCUL2 were injected into nude mice, and the cells proliferated for 4 weeks. Tumor xenograft data indicated that high levels of circCUL2 significantly decreased xenograft tumor growth and sensitized cells to cisplatin treatment (Fig. 6j-k, Additional file 2: Fig. S9E). qRT-PCR showed that the mRNA levels of circCUL2 and ROCK2 were significantly induced by circCUL2 upregulation, while the miR-142-3p level was inhibited in xenograft samples (Fig. 6l). IHC analysis of tumor xenograft samples further indicated that the protein levels of ROCK2 were notably increased upon circCUL2 overexpression (Additional file 2: Fig. S9F). These data reveal that overexpressed circCUL2 promotes cisplatin sensitivity by sponging miR-142-3p.

### circCUL2 inhibits autophagy by targeting miR-142-3p in GC cells resistant to cisplatin

**Fig. 7:**
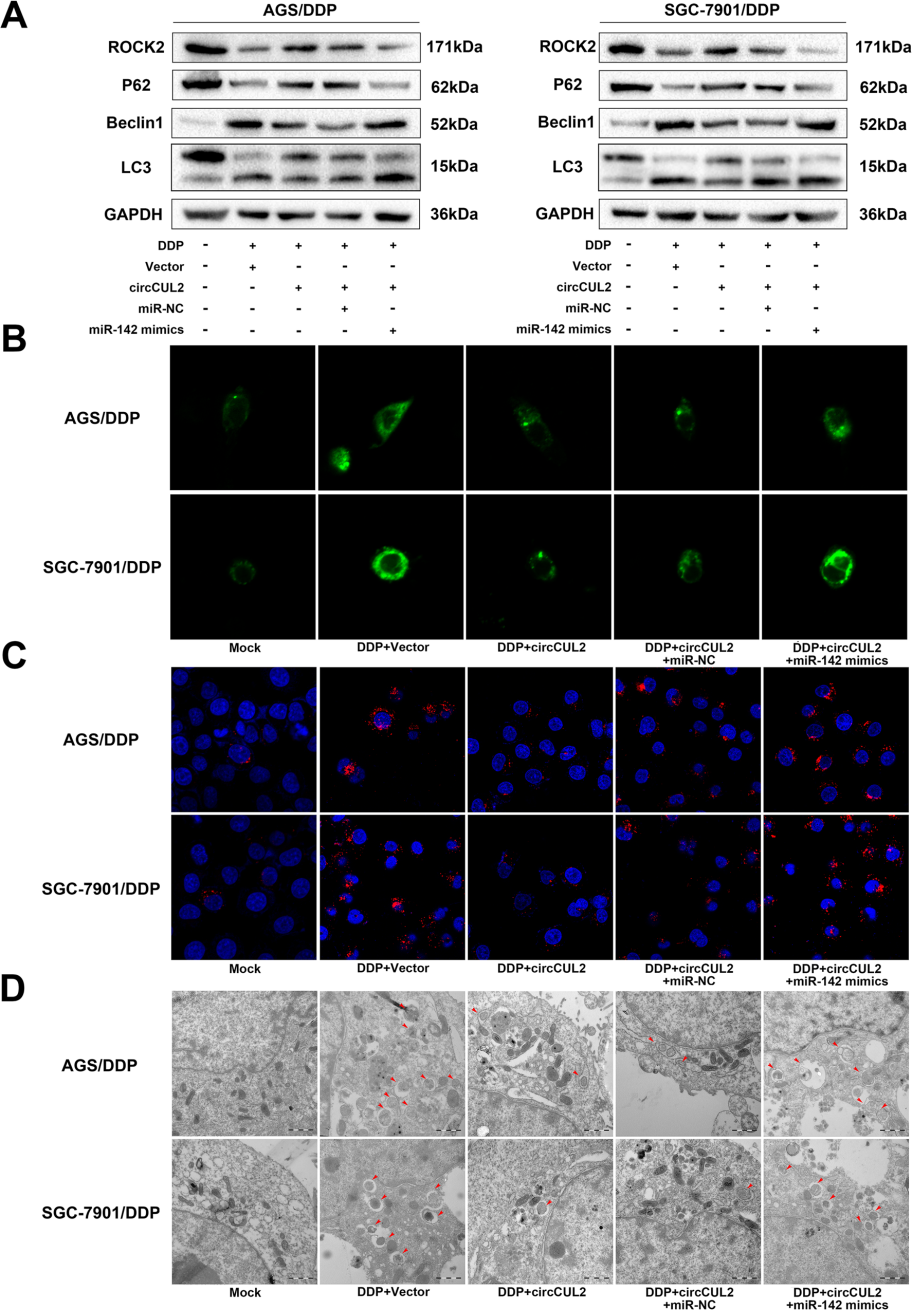
circCUL2 activates autophagy by targeting miR-142-3p in cisplatin-resistant GC cells. Cisplatin-resistant AGS and SGC-7901 cells (AGS/DDP and SGC-7901/DDP) were treated with the following conditions: mock, DDP+ vector, DDP+ circCUL2 overexpression plasmid, DDP+ circCUL2+ miR-NC, and DDP+ circCUL2+ miR-142-3p mimics. **a**: Western blot analysis was used to detect ROCK2 and autophagy-related proteins (P62, Beclin 1 and LC3 A/B). **b**: Cells were transfected with the GFP-LC3B plasmid for 24 h to detect autophagy activation. **c**: Immunofluorescence (IF) was used to detect the activation of autophagy in all cells. **d**: Electron microscopy was used to view the autophagosome number in cisplatin-resistant cells

Autophagy, which is related to differentially expressed miR-142-3p and ROCK2, plays an essential role in circRNA-regulated cancer progression and chemoresistance [19–21]. As circCUL2 elevates cisplatin sensitivity in GC, it is interesting to investigate whether circCUL2 inhibits autophagy through miR-142-3p in the AGS/DDP and SGC-7901/DDP cell lines. As expected, western blot analysis revealed that ROCK2 and p62 expression was increased by circCUL2 overexpression and was rescued by miR-142-3p mimics (Fig. 7a). Moreover, circCUL2 suppressed LC3 and Beclin1, which are markers of autophagy activity, but this effect was reversed when miR-142-3p was overexpressed in cisplatin-resistant GC cells (Fig. 7a). Cells transfected to change the expression of circCUL2 and miR-142-3p were further treated with cisplatin to analyze the autophagic activity of AGS/DDP and SGC-7901/DDP cells. As expected, compared with the mock cells, cells pretreated with an exogenous GFP-LC3B plasmid in the DDP-treated cells showed a significant increase in GFP-LC3B puncta, indicating the formation of autophagic vesicles (Fig. 7b). Moreover, cells overexpressing circCUL2 had less formation of autophagic vesicles than vector-treated cells. Moreover, the decrease in autophagic vesicle formation could be reversed by miR-142-3p mimics (Fig. 7b). Similarly, the LC3 protein expression (Fig. 7c) calculated by immunofluorescence and autophagosome number (Fig. 7d) and viewed by transmission electron microscopy had the same trend as the GFP-LC3B plasmid transfection results. Hence, these results suggest that circCUL2 inhibits autophagy via miR-142-3p/ROCK2 in cisplatin-resistant GC cells.

## Discussion

Recently, circRNAs have been proven to be differentially expressed in various diseases, especially cancers, due to their stability, abundance, conservation, and spatiotemporal specificity [22–25]. Currently, many studies have detected the differential expression of circRNAs in GC tissues [26–28]. In this study, we found that circCUL2 was downregulated in both GC tissues and GC cell lines, which suggested that circCUL2 may act as a factor to regulate GC progression. In addition, circRNAs are involved in all kinds of physiological and pathological processes, including proliferation, invasion, migration and chemoresistance, in GC development and treatment [29, 30]. Here, we revealed that circCUL2 overexpression inhibited GC cell proliferation, migration, invasion and chemoresistance in vitro. Xenograft experiments confirmed these results in vivo. However, in hepatocellular carcinoma, circCUL2 was found to promote epithelial-mesenchymal transition (EMT), tumor metastasis and malignancy [31]. The difference in expression and function of circCUL2 may be related to the tissue specificity of circRNA.

Accumulating evidence indicates that circRNAs could be miRNA sponges [7, 32]. circRNAs always play functional roles as tumor regulators by binding to miRNAs as competing endogenous RNAs (ceRNAs), therefore modulating miRNA target gene expression. circRHOBTB3 might function as a ceRNA for miR-654-3p to inhibit the growth of GC by activating the p21 signaling pathway [33]. In our study, miR-142-3p was selected as the candidate target miRNA of circCUL2 through bioinformatics analysis. A study integrating 4 GEO datasets (GSE93415, GSE23739, GSE28700, and GSE26645) showed that miR-142-3p was significantly upregulated in GC tissues [34]. In addition, miR-142-3p expression in gastric MALT lymphoma was significantly increased compared with that in chronic gastritis, consistent with the results of this study [35]. However, the role of miR-142-3p in tumors is still unclear, as miR-142-3p was downregulated in GC samples in some studies [36, 37]. In prostate cancer, reducing miR-142-3p expression can significantly inhibit cell proliferation and induce cell cycle arrest [38]. Herein, miR-142-3p was upregulated in GC tissues and promoted tumor metastasis and malignancy. The GC samples used in different studies may have different origins or differ in tissue structure. Furthermore, the miR-142-3p may act on different targets and play different roles in GC cells with different degrees of differentiation. FISH assays were then conducted to verify the colocalization in the cytoplasm and opposite expression of circCUL2 and miR-142-3p. In addition, luciferase reporter, RNA pull-down and RIP assays confirmed the direct interaction of circCUL2 and miR-142-3p. Furthermore, ROCK2, a serine-threonine kinase that can act on the cytoskeleton to regulate the morphology and migration ability of cells [39], was predicted as a miR-142-3p target gene and was confirmed by dual-luciferase reporter assays. In colorectal cancer, the inhibition of ROCK2 expression triggers the initial polarization of the colon cancer cell line and induces cell invasion [40]. In this study, ROCK2 was downregulated in GC tissues, and the correlation of circCUL2, miR-142-3p and ROCK2 expression was also tested in vivo and in vitro.

Cisplatin treatment is one of the most predominant chemotherapeutic strategies for patients with GC [41], and chemoresistance is the main reason leading to poor prognosis of GC patients [42]. In recent studies, circRNA plays a vital role in the regulation of GC cell cisplatin sensitivity. circFN1, which is increased in cisplatin-resistant GC tissues and cells, promotes the proliferation and inhibits the apoptosis of GC cells exposed to cisplatin in vivo and vitro [43]. Our study showed that circCUL2 regulated cisplatin sensitivity both in vivo and in vitro. Analysis of the TCGA database showed that expression of miR-142-3p and ROCK2 was significantly correlated with the survival time of GC patients after chemotherapy, which suggested that miR-142-3p and ROCK2 may also be involved in chemotherapeutic resistance in GC. Studies have analyzed the miRNA profiles of chemo-sensitive and chemo-resistant GC samples and found that miR-142-3p was significantly upregulated in chemotherapy-resistant GC patients [17]. In breast cancer cells, the downregulation of ROCK2 changed the hardness of the extracellular matrix, which affects the chemosensitivity of tumor cells [44]. However, in pancreatic cancer and colon cancer, ROCK2 can promote the resistance of tumor cells to chemotherapy [45, 46]. This research revealed that circCUL2 may affect the cisplatin sensitivity of GC cells by regulating miR-142-3p/ROCK2 by cotransfecting circCUL2 overexpression plasmids and miR-142-3p mimics.

Autophagy plays an important role in the mechanism of chemoresistance in GC cells. Abnormally activated autophagy induced by chemotherapeutic drugs could provide energy to support cancer cells, thereby promoting chemotherapeutic resistance [47]. Previous studies have shown that circRNA regulates downstream target genes through the mechanism of ceRNA to mediate autophagy and participate in the regulation of drug resistance in GC cells [48]. In addition, during cardiac fibrosis, ROCK2 knockout promotes age-related or starvation-induced autophagy activation [21]. Moreover, ROCK2 inhibition can promote autophagy and reduce hippocampal damage during subarachnoid hemorrhage (SAH) [49]. Intriguingly, the results of this study showed that ROCK2 was significantly increased in cisplatin-resistant cells overexpressing circCUL2, and ROCK2 might be the key mechanism by which circCUL2 regulates autophagy and drug resistance in GC cells through miR-142-3p.

## Conclusion

In summary, circCUL2 was downregulated in GC tissues and cell lines, and decreased circCUL2 expression was correlated with the TNM stage, tumor differentiation, and lymphatic metastasis of GC patients. Overexpression of circCUL2 inhibited cell proliferation, migration and invasion, probably via sponging of miR-142-3p to regulate ROCK2 in vitro and in vivo. Moreover, circCUL2 regulated cisplatin sensitivity through miR-142-3p/ROCK2-induced autophagy. In summary, circCUL2/miR-142-3p/ROCK2 may be the key mechanism and a therapeutic target for GC.

## Supplementary information

**Additional file 1: Table S1.** siRNA and RNA oligonucleotides sequences. **Table S2.** The primer sequence of qRT-PCR.

**Additional file 2: Figure S1.** A: Heatmap of circRNA sequencing (GSE100170); B: Volcano plot of GSE100170. C and D: GO (C) and KEGG (D) analyses of differentially expressed circRNA host genes. **Figure S2.** A-I: The associations of circCUL2 expression with clinical stage (A), lymph node status (B), histological type (C), gender (D), age (E), tumor size (F), tumor depth (G), *Helicobacter pylori* infection status (H) and Lauren classification (I) as determined through qRT-PCR. **Figure S3.** A: Schematic representation and target sequences of the siRNAs specific to the backsplice junction of circCUL2. B-C: The proliferation of SGC-7901 cells transfected with circCUL2-specific siRNA or an overexpression plasmid was assessed by EdU (B) and colony formation assays (C). D: Wound healing assay to assess the effect of circCUL2 on cell migration. E: Transwell assay to assess the migration and invasion of SGC-7901 cells. **Figure S4.** A: The mRNA expression of CUL2 was significantly increased in cells transfected with the pcDNA-3.1 CUL2 vector (pcDNA3.1-CUL2) and decreased in cells transfected with CUL2 siRNA. B and C: The proliferation of GC cells transfected with pcDNA3.1-CUL2 and CUL2 siRNA was assessed by CCK-8 (B) and EdU (C) assays. D: Overexpression and knockdown of CUL2 did not change the migration and invasion capacities of GC cells. **Figure S5.** A: The protein level of ROCK2 in GC tissues was evaluated by IHC. B-H: The association of circCUL2 expression with clinical stage (B), lymph node status (C), histological type (D), gender (E), age (F), tumor size (G) and tumor depth (H). I: The mRNA levels of circCUL2, miR-142-3p and ROCK2 in mouse tumor tissues. J: The ROCK2 protein level in mouse tumor tissues, as evaluated by IHC. **Figure S6.** A: circCUL2, miR-142-3p and ROCK2 expression in AGS and SGC-7901 cells with circCUL2 overexpression or knockdown. B: miR-142-3p and ROCK2 expression in AGS and SGC-7901 cells transfected with miR-142-3p mimics or an inhibitor. C: Luciferase reporter assay was used to detect the binding of miR-142-3p to circCUL2 and ROCK2 in SGC-7901 cell lines. D: qRT-PCR of circCUL2 and miR-142-3p expression pulled down from SGC-7901 cell lysates and enriched with a circCUL2-specific probe. E-F: Cotransfection of miR-142-3p mimics and circCUL2 overexpression plasmids or miR-142-3p inhibitors and circCUL2-specific siRNA to detect the mRNA (E) and protein (F) levels of ROCK2 in SGC-7901 cell lines. G-K: Cotransfection of miR-142-3p mimics and circCUL2 overexpression plasmids to investigate malignant transformation by CCK-8 (G), EdU (H), colony formation (I), wound healing (J) and Transwell (K) assays in the SGC-7901 cell line. **Figure S7.** A and B: The miR-142-3p (A) and ROCK2 (B) levels in GC patients from TCGA database. C: The negative correlation of miR-142-3p with ROCK2 based on TCGA data (*P* < 0.01). D-E: The association of miR-142-3p (D) and ROCK2 (E) expression with the clinical stage. F: The mRNA expression of ROCK2 in cells cotransfected with miR-142-3p inhibitors and ROCK2 siRNA. G and H: The proliferation, migration and invasion of cells cotransfected with miR-142-3p inhibitors and ROCK2 siRNA were measured by CCK-8 (G), EdU (H) and Transwell assays(I). **Figure S8.** A-C: Relative viability of circCUL2 overexpression plasmid- or siRNA-transfected cells exposed to MMC (A), DOX (B) and 5-FU (C) at the indicated concentrations for 48 h. **Figure S9.** A-D: Cell growth (A), IC50 values (B), and apoptosis (C and D) in SGC-7901/DDP cell lines cotransfected with miR-142-3p mimics and circCUL2 overexpression plasmids. E: mRNA levels of circCUL2, miR-142-3p and ROCK2 in mouse tumor tissues. F: ROCK2 protein levels in mouse tumor tissues, as evaluated by IHC.

## Data Availability

All data generated or analyzed during this study are included in this published article and its Additional Files.

## Abbreviations

5-FU: 5-Fluorouracil
CCK-8: Cell counting kit-8
circRNA: Circular RNA
DAPI: 4′,6-Diamidino-2-phenylindole
DDP: Cisplatin
DOX: Doxorubicin hydrochloride
EdU: 5-Ethynyl-2′-deoxyuridine
FBS: Fetal bovine serum
GC: Gastric cancer
GO: Gene Ontology
KEGG: Kyoto Encyclopedia of Genes and Genomes
miRNA: MicroRNA
MMC: Mitomycin C
OD: Optical density
qRT-PCR: Quantitative real-time polymerase chain reaction
RIPA: Radioimmunoprecipitation assay
RNase R: Ribonuclease R
ROCK2: Recombinant Rho associated coiled-coil–containing protein kinase 2
WB: Western blot
