# Supplementary material for: circCUL2 regulates gastric cancer malignant transformation and cisplatin resistance by modulating autophagy activation via miR-142-3p/ROCK2
Source: Mol Cancer. 2020 Nov 5;19:156. doi: 10.1186/s12943-020-01270-x (PMC7643398; doi:10.1186/s12943-020-01270-x)
Supplement: Supplementary file 1 — Additional file 1: Table S1. siRNA and RNA oligonucleotides sequences. Table S2. The primer sequence of qRT-PCR. [file 12943_2020_1270_MOESM1_ESM.docx]

Table S1: siRNA and RNA oligonucleotides sequences.

| 名称 | Sense（5’-3’） |
| --- | --- |
| si1-circCUL2 | GGACUGCUUAUAUAGAUUUCA |
| si2-circCUL2 | UGGACUGCUUAUAUAGAUUUC |
| si-Control | UAAGGCUAUGAAGAGAUAC |
| miR-142-3p mimics | UGUAGUGUUUCCUACUUUAUGGA |
| mimics control | UUCUCCGAACGUGUCACGUTT |
| miR-142-3p inhibitor | UCCAUAAAGUAGGAAACACUACA |
| Inhibitor control | CAGUACUUUUGUGUAGUACAA |

| circCUL2 | F: 5’-AGAATACAGCAAGGGTGCAGA-3’  R: 5’-ACCACGGCTTTTATTGTCGT-3’ |
| --- | --- |
| ITGAV | F: 5’-GCTGTCGGAGATTTCAATGGT-3’ |
|  | R: 5’-TCTGCTCGCCAGTAAAATTGT-3’ |
| MYLK | F: 5’-CCCGAGGTTGTCTGGTTCAAA-3’ |
|  | R: 5’-GCAGGTGTACTTGGCATCGT-3’ |
| CLTA | F:5’-TCCAACAGACAGTTATGCAGC-3’  R:5’-CCATTTACGGATACTTTCAGGCT-3’ |
| CFL2 | F:5’-AGAGGACCCCTACACATCTTTT-3’  R:5’-CCAAGCCATTTACTTGCCACTC-3’ |
| MYH10 | F:5’-TGGTTTTGAGGCAGCTAGTATCA-3’  R:5’-AGTCCTGAATAGTAGCGATCCTT-3’ |
| ARNTL | F:5’-AAGGGAAGCTCACAGTCAGAT-3’  R:5’-GGACATTGCGTTGCATGTTGG-3’ |
| TAOK1 | F:5’-ATGCCATCAACTAACAGAGCAG-3’  R:5’-CGCACATCTCGTGCAAAATAC-3’ |
| STX12 | F:5’-CATCCAGCGGATCAGCCAAG-3’  R:5’-GGAGTGTTGTAACTGTTGCAGAT-3’ |
| ROCK2 | F:5’-TCAGAGGTCTACAGATGAAGGC-3’ |
|  | R:5’-CCAGGGGCTATTGGCAAAGG-3’ |
| GAPDH | F:5’-GCACCGTCAAGGCTGAGAAC-3’  R:5’-GGATCTCGCTCCTGGAAGATG-3’ |

Table S2: The primer sequence of qRT-PCR
