# Supplementary figures and images for: circCUL2 regulates gastric cancer malignant transformation and cisplatin resistance by modulating autophagy activation via miR-142-3p/ROCK2
Source: Mol Cancer. 2020 Nov 5;19:156. doi: 10.1186/s12943-020-01270-x (PMC7643398; doi:10.1186/s12943-020-01270-x)

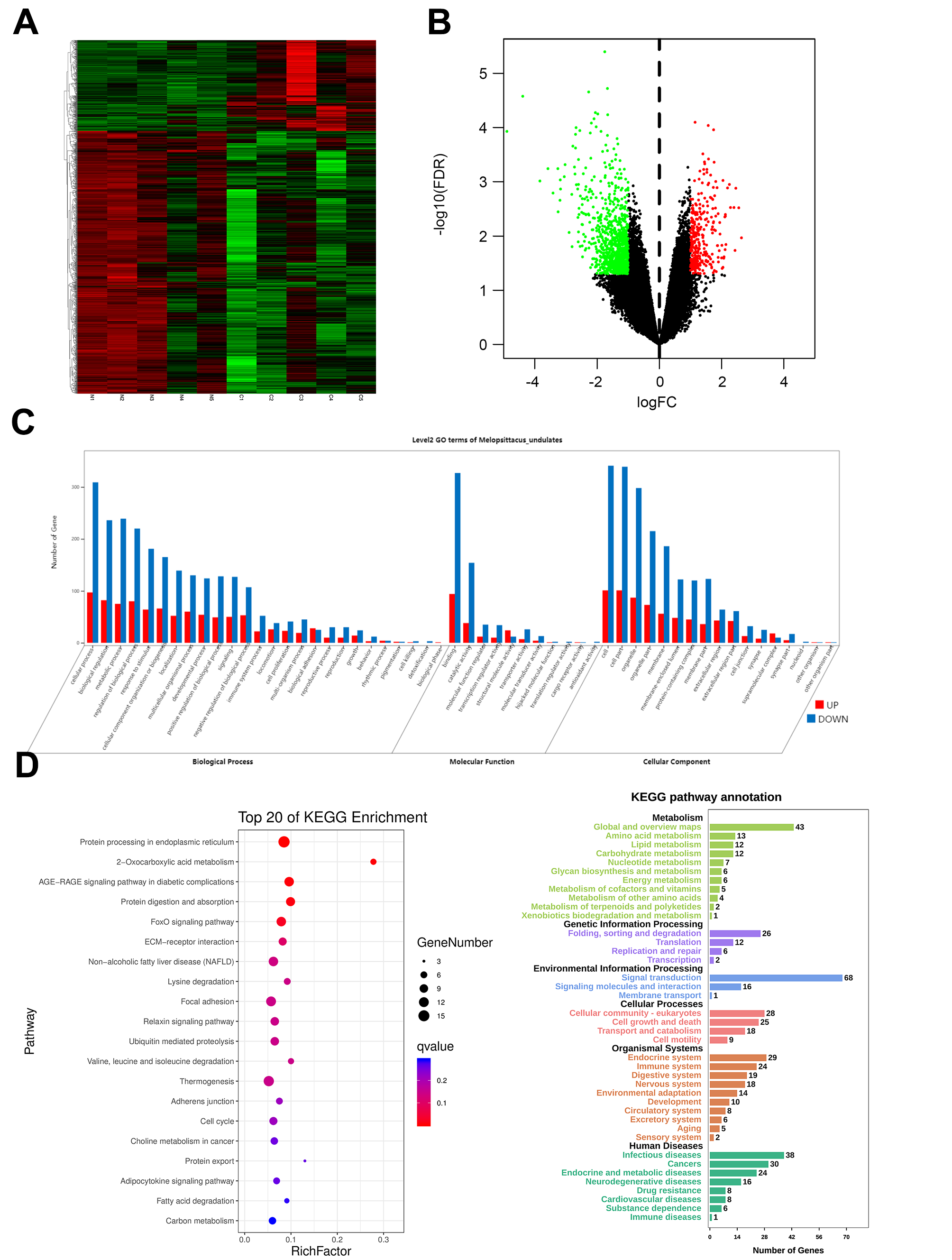

Supplement: Supplementary file 2 — Additional file 2: Figure S1. A: Heatmap of circRNA sequencing (GSE100170); B: Volcano plot of GSE100170. C and D: GO (C) and KEGG (D) analyses of differentially expressed circRNA host genes. Figure S2. A-I: The associations of circCUL2 expression with clinical stage (A), lymph node status (B), histological type (C), gender (D), age (E), tumor size (F), tumor depth (G), Helicobacter pylori infection status (H) and Lauren classification (I) as determined through qRT-PCR. Figure S3. A: Schematic representation and target sequences of the siRNAs specific to the backsplice junction of circCUL2. B-C: The proliferation of SGC-7901 cells transfected with circCUL2-specific siRNA or an overexpression plasmid was assessed by EdU (B) and colony formation assays (C). D: Wound healing assay to assess the effect of circCUL2 on cell migration. E: Transwell assay to assess the migration and invasion of SGC-7901 cells. Figure S4. A: The mRNA expression of CUL2 was significantly increased in cells transfected with the pcDNA-3.1 CUL2 vector (pcDNA3.1-CUL2) and decreased in cells transfected with CUL2 siRNA. B and C: The proliferation of GC cells transfected with pcDNA3.1-CUL2 and CUL2 siRNA was assessed by CCK-8 (B) and EdU (C) assays. D: Overexpression and knockdown of CUL2 did not change the migration and invasion capacities of GC cells. Figure S5. A: The protein level of ROCK2 in GC tissues was evaluated by IHC. B-H: The association of circCUL2 expression with clinical stage (B), lymph node status (C), histological type (D), gender (E), age (F), tumor size (G) and tumor depth (H). I: The mRNA levels of circCUL2, miR-142-3p and ROCK2 in mouse tumor tissues. J: The ROCK2 protein level in mouse tumor tissues, as evaluated by IHC. Figure S6. A: circCUL2, miR-142-3p and ROCK2 expression in AGS and SGC-7901 cells with circCUL2 overexpression or knockdown. B: miR-142-3p and ROCK2 expression in AGS and SGC-7901 cells transfected with miR-142-3p mimics or an inhibitor. C: Luciferase reporte [file 12943_2020_1270_MOESM2_ESM.zip › S1.tif]

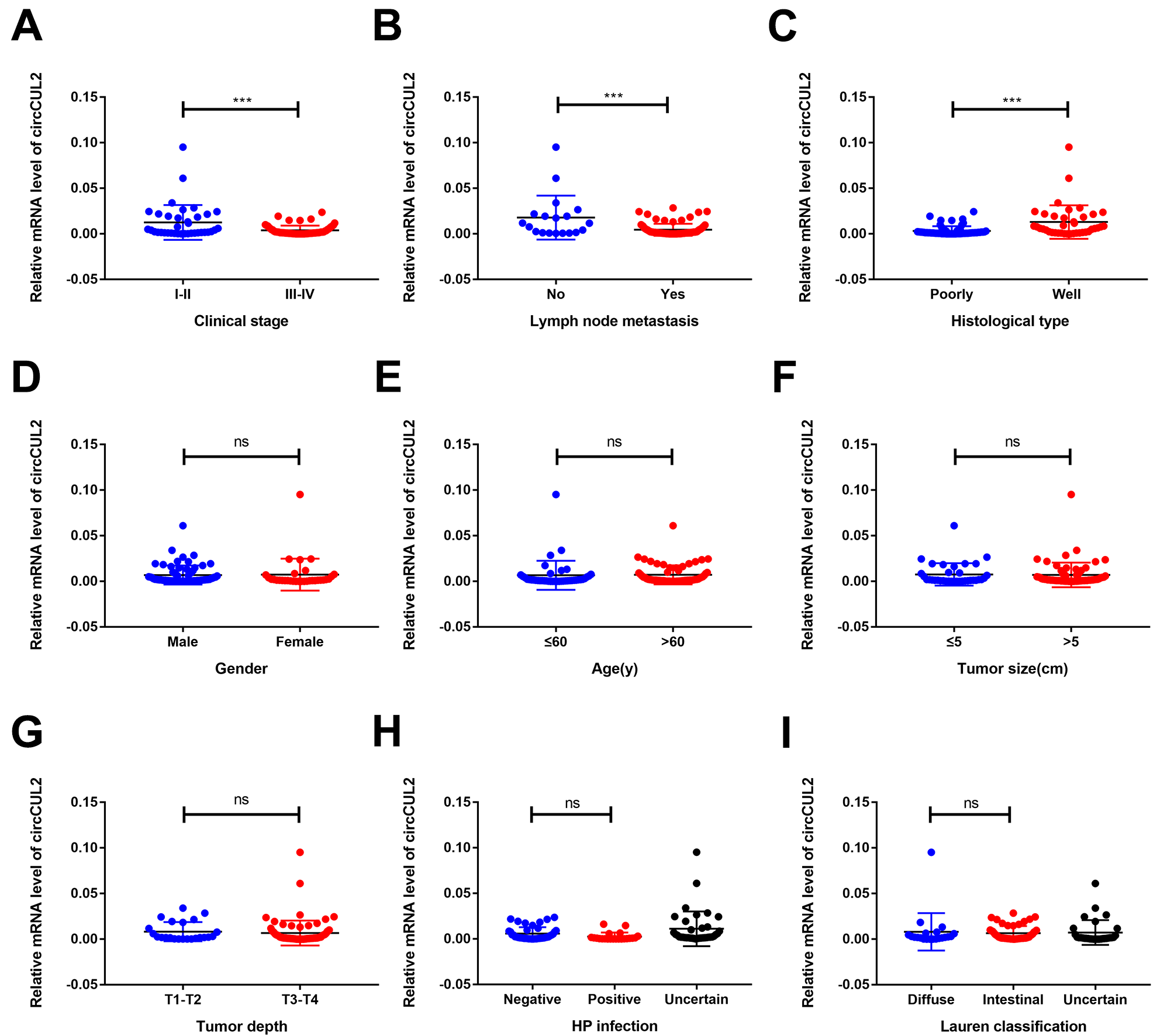

Supplement: Supplementary file 2 — Additional file 2: Figure S1. A: Heatmap of circRNA sequencing (GSE100170); B: Volcano plot of GSE100170. C and D: GO (C) and KEGG (D) analyses of differentially expressed circRNA host genes. Figure S2. A-I: The associations of circCUL2 expression with clinical stage (A), lymph node status (B), histological type (C), gender (D), age (E), tumor size (F), tumor depth (G), Helicobacter pylori infection status (H) and Lauren classification (I) as determined through qRT-PCR. Figure S3. A: Schematic representation and target sequences of the siRNAs specific to the backsplice junction of circCUL2. B-C: The proliferation of SGC-7901 cells transfected with circCUL2-specific siRNA or an overexpression plasmid was assessed by EdU (B) and colony formation assays (C). D: Wound healing assay to assess the effect of circCUL2 on cell migration. E: Transwell assay to assess the migration and invasion of SGC-7901 cells. Figure S4. A: The mRNA expression of CUL2 was significantly increased in cells transfected with the pcDNA-3.1 CUL2 vector (pcDNA3.1-CUL2) and decreased in cells transfected with CUL2 siRNA. B and C: The proliferation of GC cells transfected with pcDNA3.1-CUL2 and CUL2 siRNA was assessed by CCK-8 (B) and EdU (C) assays. D: Overexpression and knockdown of CUL2 did not change the migration and invasion capacities of GC cells. Figure S5. A: The protein level of ROCK2 in GC tissues was evaluated by IHC. B-H: The association of circCUL2 expression with clinical stage (B), lymph node status (C), histological type (D), gender (E), age (F), tumor size (G) and tumor depth (H). I: The mRNA levels of circCUL2, miR-142-3p and ROCK2 in mouse tumor tissues. J: The ROCK2 protein level in mouse tumor tissues, as evaluated by IHC. Figure S6. A: circCUL2, miR-142-3p and ROCK2 expression in AGS and SGC-7901 cells with circCUL2 overexpression or knockdown. B: miR-142-3p and ROCK2 expression in AGS and SGC-7901 cells transfected with miR-142-3p mimics or an inhibitor. C: Luciferase reporte [file 12943_2020_1270_MOESM2_ESM.zip › S2.tif]

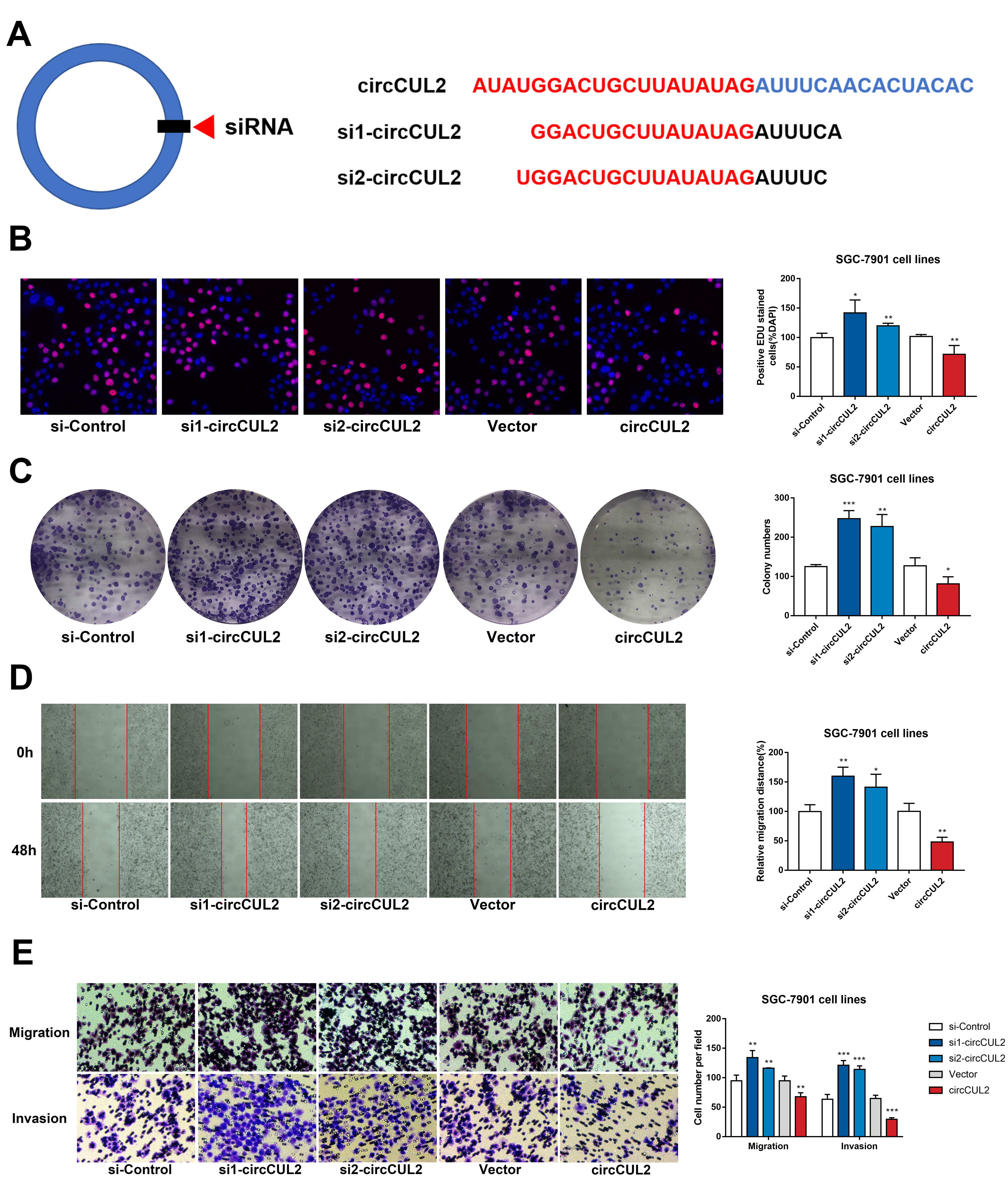

Supplement: Supplementary file 2 — Additional file 2: Figure S1. A: Heatmap of circRNA sequencing (GSE100170); B: Volcano plot of GSE100170. C and D: GO (C) and KEGG (D) analyses of differentially expressed circRNA host genes. Figure S2. A-I: The associations of circCUL2 expression with clinical stage (A), lymph node status (B), histological type (C), gender (D), age (E), tumor size (F), tumor depth (G), Helicobacter pylori infection status (H) and Lauren classification (I) as determined through qRT-PCR. Figure S3. A: Schematic representation and target sequences of the siRNAs specific to the backsplice junction of circCUL2. B-C: The proliferation of SGC-7901 cells transfected with circCUL2-specific siRNA or an overexpression plasmid was assessed by EdU (B) and colony formation assays (C). D: Wound healing assay to assess the effect of circCUL2 on cell migration. E: Transwell assay to assess the migration and invasion of SGC-7901 cells. Figure S4. A: The mRNA expression of CUL2 was significantly increased in cells transfected with the pcDNA-3.1 CUL2 vector (pcDNA3.1-CUL2) and decreased in cells transfected with CUL2 siRNA. B and C: The proliferation of GC cells transfected with pcDNA3.1-CUL2 and CUL2 siRNA was assessed by CCK-8 (B) and EdU (C) assays. D: Overexpression and knockdown of CUL2 did not change the migration and invasion capacities of GC cells. Figure S5. A: The protein level of ROCK2 in GC tissues was evaluated by IHC. B-H: The association of circCUL2 expression with clinical stage (B), lymph node status (C), histological type (D), gender (E), age (F), tumor size (G) and tumor depth (H). I: The mRNA levels of circCUL2, miR-142-3p and ROCK2 in mouse tumor tissues. J: The ROCK2 protein level in mouse tumor tissues, as evaluated by IHC. Figure S6. A: circCUL2, miR-142-3p and ROCK2 expression in AGS and SGC-7901 cells with circCUL2 overexpression or knockdown. B: miR-142-3p and ROCK2 expression in AGS and SGC-7901 cells transfected with miR-142-3p mimics or an inhibitor. C: Luciferase reporte [file 12943_2020_1270_MOESM2_ESM.zip › S3.tif]

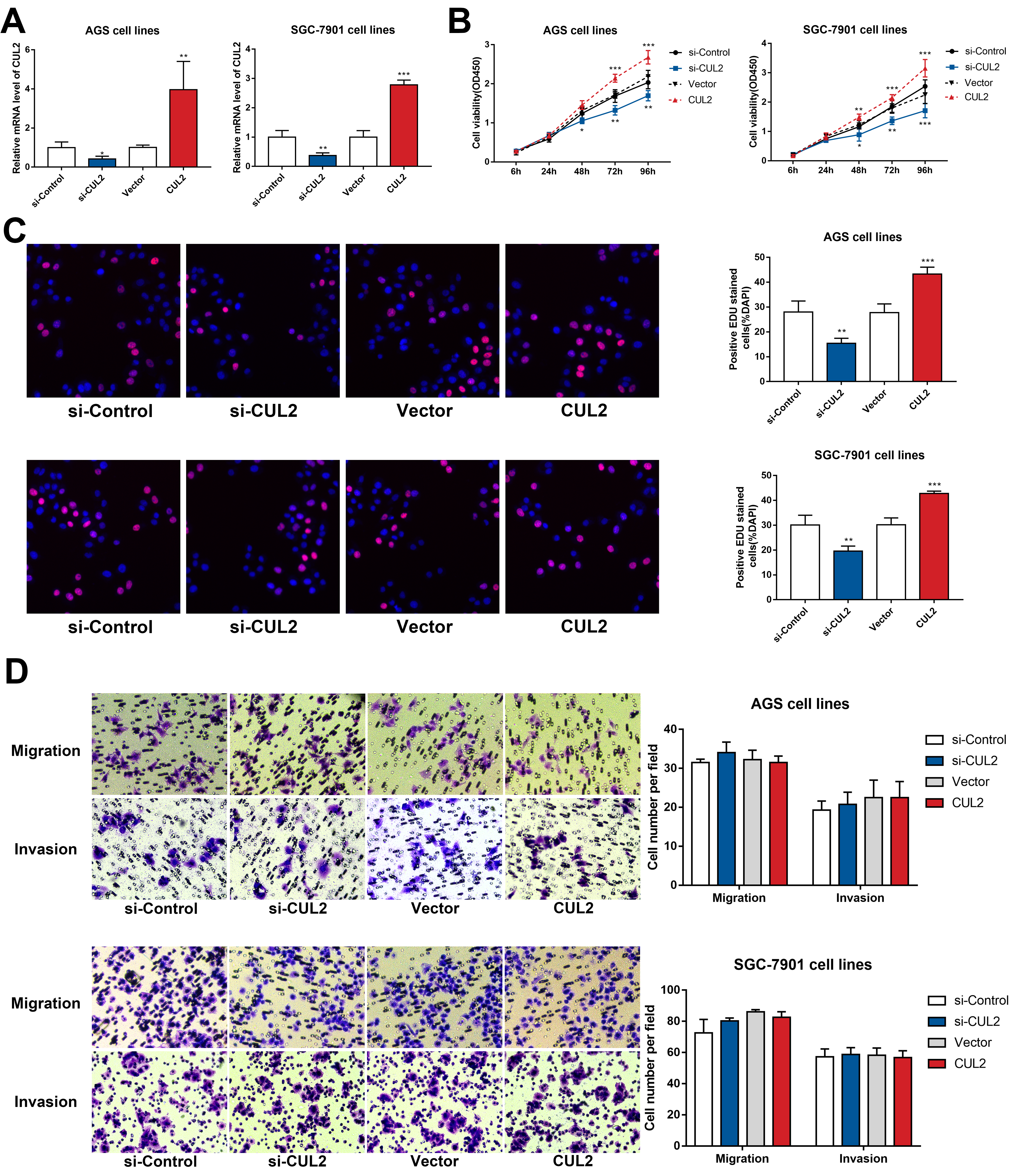

Supplement: Supplementary file 2 — Additional file 2: Figure S1. A: Heatmap of circRNA sequencing (GSE100170); B: Volcano plot of GSE100170. C and D: GO (C) and KEGG (D) analyses of differentially expressed circRNA host genes. Figure S2. A-I: The associations of circCUL2 expression with clinical stage (A), lymph node status (B), histological type (C), gender (D), age (E), tumor size (F), tumor depth (G), Helicobacter pylori infection status (H) and Lauren classification (I) as determined through qRT-PCR. Figure S3. A: Schematic representation and target sequences of the siRNAs specific to the backsplice junction of circCUL2. B-C: The proliferation of SGC-7901 cells transfected with circCUL2-specific siRNA or an overexpression plasmid was assessed by EdU (B) and colony formation assays (C). D: Wound healing assay to assess the effect of circCUL2 on cell migration. E: Transwell assay to assess the migration and invasion of SGC-7901 cells. Figure S4. A: The mRNA expression of CUL2 was significantly increased in cells transfected with the pcDNA-3.1 CUL2 vector (pcDNA3.1-CUL2) and decreased in cells transfected with CUL2 siRNA. B and C: The proliferation of GC cells transfected with pcDNA3.1-CUL2 and CUL2 siRNA was assessed by CCK-8 (B) and EdU (C) assays. D: Overexpression and knockdown of CUL2 did not change the migration and invasion capacities of GC cells. Figure S5. A: The protein level of ROCK2 in GC tissues was evaluated by IHC. B-H: The association of circCUL2 expression with clinical stage (B), lymph node status (C), histological type (D), gender (E), age (F), tumor size (G) and tumor depth (H). I: The mRNA levels of circCUL2, miR-142-3p and ROCK2 in mouse tumor tissues. J: The ROCK2 protein level in mouse tumor tissues, as evaluated by IHC. Figure S6. A: circCUL2, miR-142-3p and ROCK2 expression in AGS and SGC-7901 cells with circCUL2 overexpression or knockdown. B: miR-142-3p and ROCK2 expression in AGS and SGC-7901 cells transfected with miR-142-3p mimics or an inhibitor. C: Luciferase reporte [file 12943_2020_1270_MOESM2_ESM.zip › S4.tif]

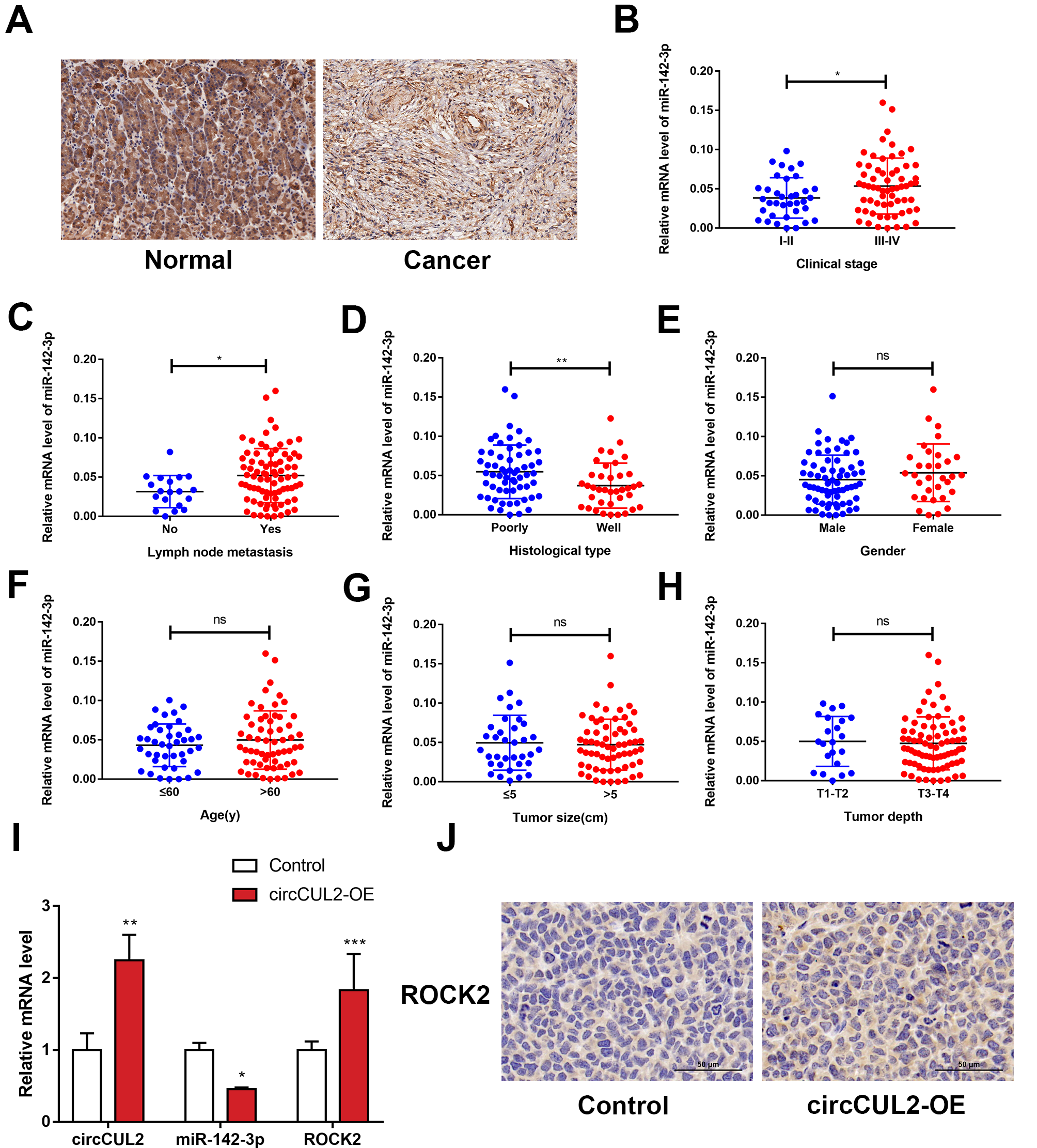

Supplement: Supplementary file 2 — Additional file 2: Figure S1. A: Heatmap of circRNA sequencing (GSE100170); B: Volcano plot of GSE100170. C and D: GO (C) and KEGG (D) analyses of differentially expressed circRNA host genes. Figure S2. A-I: The associations of circCUL2 expression with clinical stage (A), lymph node status (B), histological type (C), gender (D), age (E), tumor size (F), tumor depth (G), Helicobacter pylori infection status (H) and Lauren classification (I) as determined through qRT-PCR. Figure S3. A: Schematic representation and target sequences of the siRNAs specific to the backsplice junction of circCUL2. B-C: The proliferation of SGC-7901 cells transfected with circCUL2-specific siRNA or an overexpression plasmid was assessed by EdU (B) and colony formation assays (C). D: Wound healing assay to assess the effect of circCUL2 on cell migration. E: Transwell assay to assess the migration and invasion of SGC-7901 cells. Figure S4. A: The mRNA expression of CUL2 was significantly increased in cells transfected with the pcDNA-3.1 CUL2 vector (pcDNA3.1-CUL2) and decreased in cells transfected with CUL2 siRNA. B and C: The proliferation of GC cells transfected with pcDNA3.1-CUL2 and CUL2 siRNA was assessed by CCK-8 (B) and EdU (C) assays. D: Overexpression and knockdown of CUL2 did not change the migration and invasion capacities of GC cells. Figure S5. A: The protein level of ROCK2 in GC tissues was evaluated by IHC. B-H: The association of circCUL2 expression with clinical stage (B), lymph node status (C), histological type (D), gender (E), age (F), tumor size (G) and tumor depth (H). I: The mRNA levels of circCUL2, miR-142-3p and ROCK2 in mouse tumor tissues. J: The ROCK2 protein level in mouse tumor tissues, as evaluated by IHC. Figure S6. A: circCUL2, miR-142-3p and ROCK2 expression in AGS and SGC-7901 cells with circCUL2 overexpression or knockdown. B: miR-142-3p and ROCK2 expression in AGS and SGC-7901 cells transfected with miR-142-3p mimics or an inhibitor. C: Luciferase reporte [file 12943_2020_1270_MOESM2_ESM.zip › S5.tif]

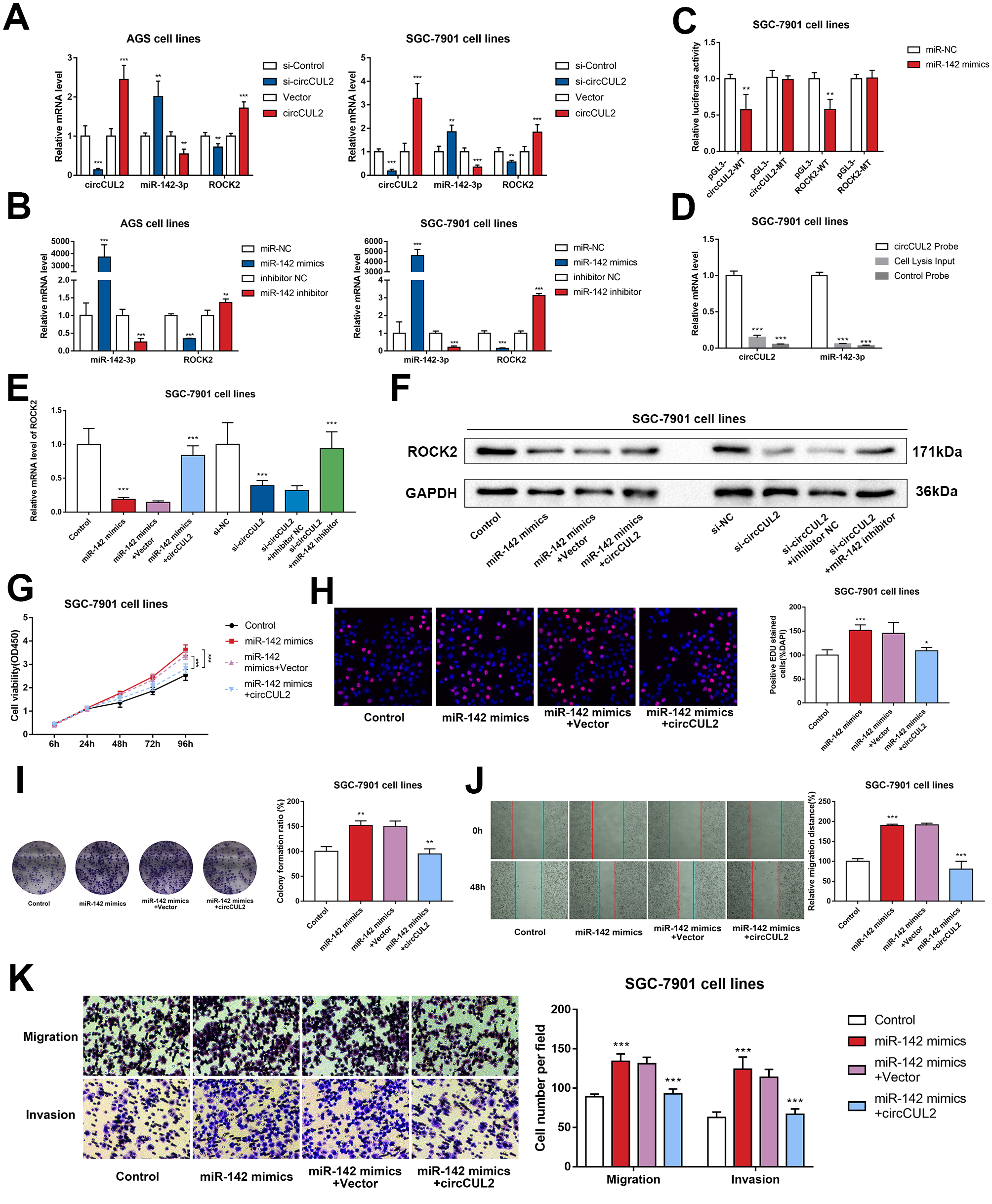

Supplement: Supplementary file 2 — Additional file 2: Figure S1. A: Heatmap of circRNA sequencing (GSE100170); B: Volcano plot of GSE100170. C and D: GO (C) and KEGG (D) analyses of differentially expressed circRNA host genes. Figure S2. A-I: The associations of circCUL2 expression with clinical stage (A), lymph node status (B), histological type (C), gender (D), age (E), tumor size (F), tumor depth (G), Helicobacter pylori infection status (H) and Lauren classification (I) as determined through qRT-PCR. Figure S3. A: Schematic representation and target sequences of the siRNAs specific to the backsplice junction of circCUL2. B-C: The proliferation of SGC-7901 cells transfected with circCUL2-specific siRNA or an overexpression plasmid was assessed by EdU (B) and colony formation assays (C). D: Wound healing assay to assess the effect of circCUL2 on cell migration. E: Transwell assay to assess the migration and invasion of SGC-7901 cells. Figure S4. A: The mRNA expression of CUL2 was significantly increased in cells transfected with the pcDNA-3.1 CUL2 vector (pcDNA3.1-CUL2) and decreased in cells transfected with CUL2 siRNA. B and C: The proliferation of GC cells transfected with pcDNA3.1-CUL2 and CUL2 siRNA was assessed by CCK-8 (B) and EdU (C) assays. D: Overexpression and knockdown of CUL2 did not change the migration and invasion capacities of GC cells. Figure S5. A: The protein level of ROCK2 in GC tissues was evaluated by IHC. B-H: The association of circCUL2 expression with clinical stage (B), lymph node status (C), histological type (D), gender (E), age (F), tumor size (G) and tumor depth (H). I: The mRNA levels of circCUL2, miR-142-3p and ROCK2 in mouse tumor tissues. J: The ROCK2 protein level in mouse tumor tissues, as evaluated by IHC. Figure S6. A: circCUL2, miR-142-3p and ROCK2 expression in AGS and SGC-7901 cells with circCUL2 overexpression or knockdown. B: miR-142-3p and ROCK2 expression in AGS and SGC-7901 cells transfected with miR-142-3p mimics or an inhibitor. C: Luciferase reporte [file 12943_2020_1270_MOESM2_ESM.zip › S6.tif]

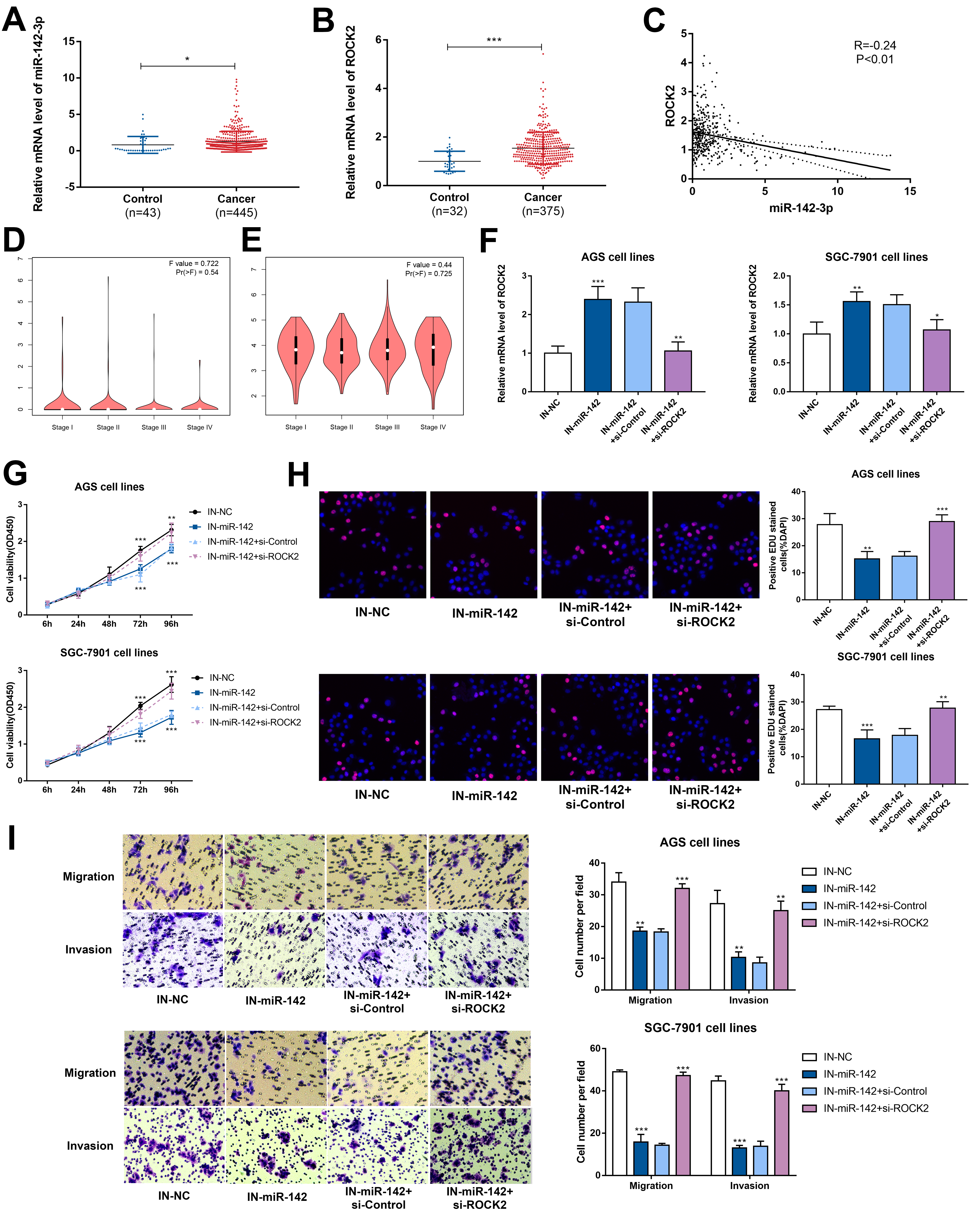

Supplement: Supplementary file 2 — Additional file 2: Figure S1. A: Heatmap of circRNA sequencing (GSE100170); B: Volcano plot of GSE100170. C and D: GO (C) and KEGG (D) analyses of differentially expressed circRNA host genes. Figure S2. A-I: The associations of circCUL2 expression with clinical stage (A), lymph node status (B), histological type (C), gender (D), age (E), tumor size (F), tumor depth (G), Helicobacter pylori infection status (H) and Lauren classification (I) as determined through qRT-PCR. Figure S3. A: Schematic representation and target sequences of the siRNAs specific to the backsplice junction of circCUL2. B-C: The proliferation of SGC-7901 cells transfected with circCUL2-specific siRNA or an overexpression plasmid was assessed by EdU (B) and colony formation assays (C). D: Wound healing assay to assess the effect of circCUL2 on cell migration. E: Transwell assay to assess the migration and invasion of SGC-7901 cells. Figure S4. A: The mRNA expression of CUL2 was significantly increased in cells transfected with the pcDNA-3.1 CUL2 vector (pcDNA3.1-CUL2) and decreased in cells transfected with CUL2 siRNA. B and C: The proliferation of GC cells transfected with pcDNA3.1-CUL2 and CUL2 siRNA was assessed by CCK-8 (B) and EdU (C) assays. D: Overexpression and knockdown of CUL2 did not change the migration and invasion capacities of GC cells. Figure S5. A: The protein level of ROCK2 in GC tissues was evaluated by IHC. B-H: The association of circCUL2 expression with clinical stage (B), lymph node status (C), histological type (D), gender (E), age (F), tumor size (G) and tumor depth (H). I: The mRNA levels of circCUL2, miR-142-3p and ROCK2 in mouse tumor tissues. J: The ROCK2 protein level in mouse tumor tissues, as evaluated by IHC. Figure S6. A: circCUL2, miR-142-3p and ROCK2 expression in AGS and SGC-7901 cells with circCUL2 overexpression or knockdown. B: miR-142-3p and ROCK2 expression in AGS and SGC-7901 cells transfected with miR-142-3p mimics or an inhibitor. C: Luciferase reporte [file 12943_2020_1270_MOESM2_ESM.zip › S7.tif]

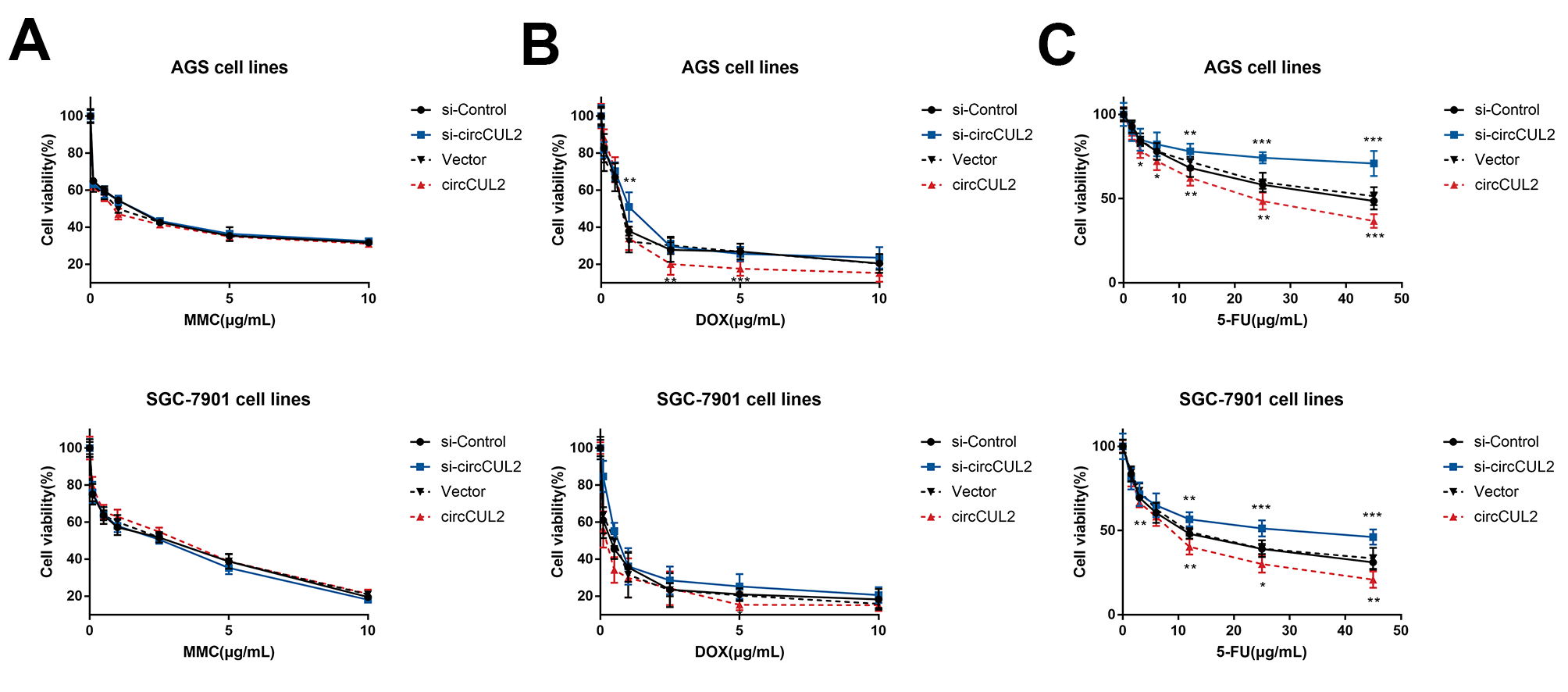

Supplement: Supplementary file 2 — Additional file 2: Figure S1. A: Heatmap of circRNA sequencing (GSE100170); B: Volcano plot of GSE100170. C and D: GO (C) and KEGG (D) analyses of differentially expressed circRNA host genes. Figure S2. A-I: The associations of circCUL2 expression with clinical stage (A), lymph node status (B), histological type (C), gender (D), age (E), tumor size (F), tumor depth (G), Helicobacter pylori infection status (H) and Lauren classification (I) as determined through qRT-PCR. Figure S3. A: Schematic representation and target sequences of the siRNAs specific to the backsplice junction of circCUL2. B-C: The proliferation of SGC-7901 cells transfected with circCUL2-specific siRNA or an overexpression plasmid was assessed by EdU (B) and colony formation assays (C). D: Wound healing assay to assess the effect of circCUL2 on cell migration. E: Transwell assay to assess the migration and invasion of SGC-7901 cells. Figure S4. A: The mRNA expression of CUL2 was significantly increased in cells transfected with the pcDNA-3.1 CUL2 vector (pcDNA3.1-CUL2) and decreased in cells transfected with CUL2 siRNA. B and C: The proliferation of GC cells transfected with pcDNA3.1-CUL2 and CUL2 siRNA was assessed by CCK-8 (B) and EdU (C) assays. D: Overexpression and knockdown of CUL2 did not change the migration and invasion capacities of GC cells. Figure S5. A: The protein level of ROCK2 in GC tissues was evaluated by IHC. B-H: The association of circCUL2 expression with clinical stage (B), lymph node status (C), histological type (D), gender (E), age (F), tumor size (G) and tumor depth (H). I: The mRNA levels of circCUL2, miR-142-3p and ROCK2 in mouse tumor tissues. J: The ROCK2 protein level in mouse tumor tissues, as evaluated by IHC. Figure S6. A: circCUL2, miR-142-3p and ROCK2 expression in AGS and SGC-7901 cells with circCUL2 overexpression or knockdown. B: miR-142-3p and ROCK2 expression in AGS and SGC-7901 cells transfected with miR-142-3p mimics or an inhibitor. C: Luciferase reporte [file 12943_2020_1270_MOESM2_ESM.zip › S8.tif]

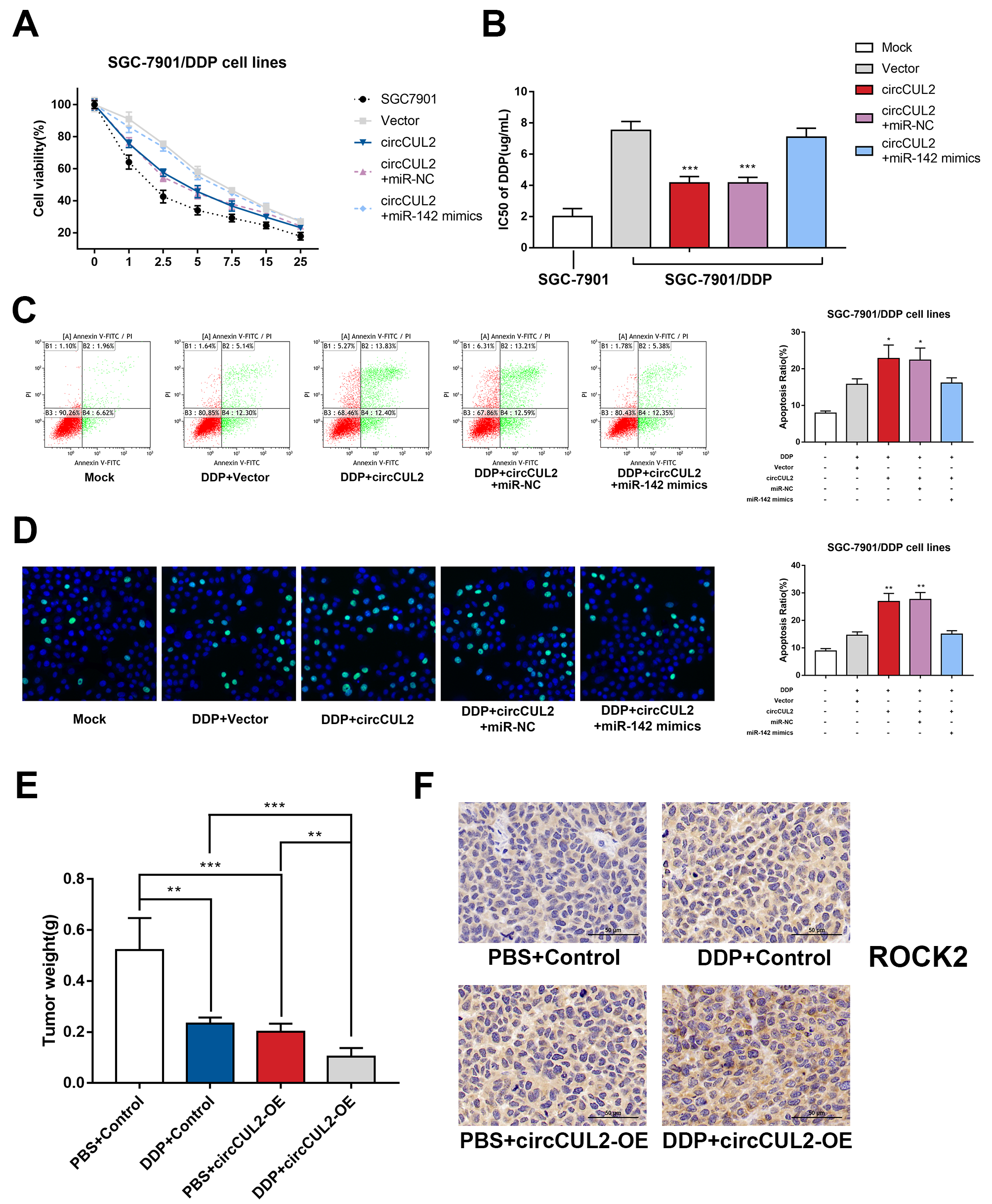

Supplement: Supplementary file 2 — Additional file 2: Figure S1. A: Heatmap of circRNA sequencing (GSE100170); B: Volcano plot of GSE100170. C and D: GO (C) and KEGG (D) analyses of differentially expressed circRNA host genes. Figure S2. A-I: The associations of circCUL2 expression with clinical stage (A), lymph node status (B), histological type (C), gender (D), age (E), tumor size (F), tumor depth (G), Helicobacter pylori infection status (H) and Lauren classification (I) as determined through qRT-PCR. Figure S3. A: Schematic representation and target sequences of the siRNAs specific to the backsplice junction of circCUL2. B-C: The proliferation of SGC-7901 cells transfected with circCUL2-specific siRNA or an overexpression plasmid was assessed by EdU (B) and colony formation assays (C). D: Wound healing assay to assess the effect of circCUL2 on cell migration. E: Transwell assay to assess the migration and invasion of SGC-7901 cells. Figure S4. A: The mRNA expression of CUL2 was significantly increased in cells transfected with the pcDNA-3.1 CUL2 vector (pcDNA3.1-CUL2) and decreased in cells transfected with CUL2 siRNA. B and C: The proliferation of GC cells transfected with pcDNA3.1-CUL2 and CUL2 siRNA was assessed by CCK-8 (B) and EdU (C) assays. D: Overexpression and knockdown of CUL2 did not change the migration and invasion capacities of GC cells. Figure S5. A: The protein level of ROCK2 in GC tissues was evaluated by IHC. B-H: The association of circCUL2 expression with clinical stage (B), lymph node status (C), histological type (D), gender (E), age (F), tumor size (G) and tumor depth (H). I: The mRNA levels of circCUL2, miR-142-3p and ROCK2 in mouse tumor tissues. J: The ROCK2 protein level in mouse tumor tissues, as evaluated by IHC. Figure S6. A: circCUL2, miR-142-3p and ROCK2 expression in AGS and SGC-7901 cells with circCUL2 overexpression or knockdown. B: miR-142-3p and ROCK2 expression in AGS and SGC-7901 cells transfected with miR-142-3p mimics or an inhibitor. C: Luciferase reporte [file 12943_2020_1270_MOESM2_ESM.zip › S9.tif]
